# EBBO: A Biomimetically Enhanced Optimization Algorithm with Multi-Stage Cooperation for Complex Engineering Applications

**DOI:** 10.3390/biomimetics11020110

**Published:** 2026-02-03

**Authors:** Xuemei Zhu, Haoyu Cai, Shirong Li, Wei Peng

**Affiliations:** 1 Experimental Training Teaching Management Department, West Anhui University, Yu’an District, Lu’an 237012, China; 2 School of Electrical and Optoelectronic Engineering, West Anhui University, Yu’an District, Lu’an 237012, China; 3 Automotive Technology College, Anhui Vocational College of Defense Technology, Yu’an District, Lu’an 237011, China; 4 School of Electronic Information and Artificial Intelligence, West Anhui University, Yu’an District, Lu’an 237012, China

**Keywords:** Beaver Behavior Optimizer, enhanced optimization algorithm, constrained optimization, engineering optimization design, metaheuristic algorithm

## Abstract

This study proposes Enhanced Beaver Behavior Optimizer (EBBO) to overcome the original BBO algorithm’s limitations in handling complex optimization problems. EBBO integrates a three-phase cooperative framework, incorporating adaptive mutation, dynamic opposition-based learning, and an risk-aware decision strategy inspired by simulated annealing. Comprehensive evaluations on the CEC 2017 and CEC 2020 benchmark suites demonstrate that EBBO significantly outperforms nine widely used algorithms (e.g., BBO, FATA, DE) in convergence accuracy, stability, and robustness, especially for high-dimensional and multimodal functions. EBBO achieves average objective value reductions of 15–50% and standard deviation reductions of 30–70% compared to the original BBO, with Wilcoxon rank-sum tests confirming statistical significance across most functions. When applied to three classical engineering design problems—step-cone pulley, pressure vessel, three-bar truss optimization, and 3D UAV path planning—EBBO consistently achieved the best or near-optimal solutions while satisfying all nonlinear constraints. The results confirm that EBBO effectively balances exploration and exploitation, offering a reliable and efficient approach for solving complex constrained optimization challenges in both benchmark and real-world engineering contexts.

## 1. Introduction

Optimization problems are ubiquitous in the design and control of complex systems in modern science and engineering. The efficiency and quality of their solutions directly impact the feasibility and cost-effectiveness of engineering practices [1]. Traditional optimization methods, such as gradient-based approaches and linear programming, often impose stringent requirements on problem continuity and convexity, making them ill-suited for addressing real-world challenges characterized by high dimensionality, nonlinearity, multimodality, and strong constraints [2]. In this context, bio-inspired intelligent optimization algorithms have emerged as mainstream tools for solving complex optimization problems [3]. Drawing inspiration from biological behaviors, ecological evolution, and physicochemical processes in nature, these algorithms construct search mechanisms featuring self-adaptation, self-organization, and distributed characteristics, effectively overcoming the bottlenecks of balancing global exploration and local exploitation inherent in traditional methods [4].

The development of bio-inspired intelligent optimization algorithms has progressed through multiple stages, forming a diverse spectrum of algorithmic families [5]. Early algorithms, such as the Genetic Algorithm (GA) [6], which mimics the selection, crossover, and mutation mechanisms of biological evolution, laid the foundation for evolutionary computation. Particle Swarm Optimization (PSO) [7], inspired by the collaborative foraging behavior of bird flocks, pioneered the paradigm of swarm intelligence optimization. With deepening research, an increasing number of bio-inspired models have been proposed: Ant Colony Optimization (ACO) [8] simulates ant colony cooperation via pheromone trails to find shortest paths; the Artificial Bee Colony (ABC) [9] algorithm emulates the division of labor among employed bees, onlooker bees, and scout bees during honey collection; the Grey Wolf Optimizer (GWO) [10] models the social hierarchy and hunting strategies of wolf packs; the Whale Optimization Algorithm (WOA) [11] is based on the bubble-net feeding behavior of humpback whales; and the Butterfly Optimization Algorithm (BOA) [12] utilizes the perception mechanism of butterflies toward floral scents for optimization.

In recent years, more complex natural phenomena and biological behaviors have been further explored: the Harris Hawks Optimization (HHO) [13] algorithm simulates the surprise pounce strategies of hawks during cooperative prey pursuit; the Dung Beetle Optimizer (DBO) [14] draws inspiration from the ball-rolling, breeding, and foraging behaviors of dung beetles; the Crayfish Optimization Algorithm (COA) [15] is based on the summer avoidance, feeding, and competition behaviors of crayfish; the Water Uptake and Transport in Plants Algorithm (WUTP) [16] mimics the water absorption mechanism of plant root systems; and the Fata Morgana Algorithm (FATA) [17] abstracts a unique optimization mechanism from the optical phenomenon of mirages. These algorithms demonstrate distinct advantages across various optimization tasks through their diverse bio-inspired mechanisms.

However, single heuristic strategies often exhibit limitations when dealing with complex and variable engineering problems, particularly in handling highly constrained, multimodal, or mixed-variable problems [18]. Issues such as premature convergence, low search efficiency, or insufficient stability frequently arise. Consequently, researchers continue to explore more efficient bio-inspired models and enhance existing algorithms by proposing various improvement strategies, including hybridization mechanisms [19], adaptive parameter adjustment [20], multi-stage collaboration [21], and integration with other optimization paradigms [22].

The comparative algorithms discussed in this paper encompass a range of representative bio-inspired optimizers. Among these, the Beaver Behavior Optimizer (BBO) [23] has garnered sustained attention due to its well-structured biological foundation. BBO simulates the intelligent behaviors observed during beaver dam construction—material collection, role division, and environmental adaptation—by modeling beavers as “Architects” and “Prospectors” to dynamically balance exploration and exploitation.

Three key reasons motivate our selection of BBO as the basis for enhancement:

1. Clear bio-mechanistic mapping: BBO’s design directly translates observable dam-building behaviors into algorithmic operators. In nature, beaver colonies exhibit structured division of labor: architect beavers stabilize the dam structure, while prospector beavers gather new materials. This role specialization enables dynamic response to environmental changes, providing a natural analog to optimization search where material collection corresponds to global exploration and structural reinforcement to local exploitation.
2. Identifiable and addressable limitations: Despite its promising framework, the standard BBO exhibits specific shortcomings: over-reliance on random updates [24], inadequate diversity preservation [25], and insufficient utilization of elite information [26]. These well-defined limitations present clear opportunities for systematic improvement.
3. Relevance to constrained engineering problems: The dam-building process inherently involves constraint handling (environmental adaptation, material limits) and iterative refinement—characteristics directly relevant to complex engineering optimization where solutions must satisfy multiple nonlinear constraints.

Based on this analysis, we propose the Enhanced Beaver Behavior Optimizer (EBBO), which retains BBO’s core biomimetic framework while introducing a multi-stage collaborative optimization mechanism [21]. Through three key enhancements—adaptive mutation [27], dynamic opposition-based learning [28], and risk-aware decision strategy supplementation [26]—EBBO addresses BBO’s limitations while preserving its biological coherence, resulting in significantly improved global search capability, convergence accuracy, and stability.

The structure of this paper is organized as follows: Section 2 provides a detailed introduction to the mathematical model and algorithmic framework of the standard Beaver Behavior Optimizer (BBO), analyzing its core mechanisms and potential shortcomings. Section 3 systematically elaborates on the improvement strategies and algorithm design of EBBO, including the theoretical foundation and implementation details of the multi-stage collaborative framework. Section 4 conducts a systematic evaluation of EBBO using authoritative test function suites such as CEC 2017 and CEC 2020, comparing its performance with nine representative algorithms, including BBO, FATA, HHO, GA, Differential Evolution algorithm (DE) [29], DBO, COA, WUTP, and WOA. Section 5 applies EBBO to four engineering optimization problems—step-cone pulley design, pressure vessel optimization, three-bar truss design, and  3D UAV path planning—to validate its effectiveness in real-world engineering scenarios. Finally, Section 6 summarizes the work presented in this paper, highlights the advantages and limitations of EBBO, and outlines future research directions. Through this comprehensive research framework, this paper aims to provide an efficient and reliable intelligent optimization tool for solving complex engineering optimization problems and to offer new insights for the design and enhancement of bio-inspired optimization algorithms.

The primary contributions of this paper are summarized as follows:

1. EBBO maintains explicit biomimetic–computational correspondence: Prospecting maps to exploration and opposition-based learning, dam-architecting maps to exploitation and elite preservation, risk-aware decision maps to adaptive mutation and probabilistic acceptance, and colony coordination maps to the multi-stage workflow with role specialization. This systematic mapping ensures that each algorithmic module corresponds directly to a biological mechanism, preserving biological coherence rather than forming a mathematical composite.
2. Novel multi-stage collaborative optimization mechanism: EBBO introduces a structured three-stage sequential framework (core BBO updates → mutation enhancement → opposition-based learning → risk-aware decision strategy) based on biological plausibility and computational synergy. This carefully designed execution sequence, where each stage prepares the population for the next, represents a significant departure from both the original BBO and other metaheuristics that apply improvements indiscriminately or in parallel.
3. Dual-benchmark comprehensive validation with statistical rigor: EBBO is rigorously evaluated on two complementary high-dimensional benchmark suites (CEC 2017 with 29 functions and CEC 2020 with 10 functions) employing different dimensionalities (30D and 20D). The validation demonstrates statistically significant superiority over nine state-of-the-art metaheuristics across convergence accuracy, stability, and robustness metrics, with Wilcoxon tests confirming performance differences beyond random variation.
4. Practical engineering applicability across diverse constrained problems: The algorithm is successfully applied to three distinct classical engineering design problems with varying characteristics: step-cone pulley (multiple nonlinear constraints), pressure vessel (mixed discrete-continuous variables), and three-bar truss (stress and deflection constraints). EBBO consistently delivers near-optimal feasible solutions with high robustness, demonstrating broad applicability rather than narrow specialization.
5. Methodological advancement in bio-inspired algorithm design: Beyond algorithmic performance, this research contributes methodology by demonstrating how explicit behavior-operator mapping enhances algorithmic transparency and provides interpretable design principles. The work illustrates a pathway from metaphorical inspiration to mechanistic implementation, addressing common criticisms of bio-inspired heuristics lacking concrete biological correspondence.

These contributions collectively advance the field of bio-inspired optimization by (1) providing a robust and efficient tool for complex constrained optimization, (2) establishing a template for principled biomimetic algorithm design with explicit behavior-operator correspondence, and (3) demonstrating the effectiveness of structured multi-stage frameworks over indiscriminate strategy combination.

## 2. Beaver Behavior Optimizer

Beaver Behavior Optimizer (BBO) [23] is a swarm intelligence optimization algorithm inspired by the natural dam-building behavior of beavers. By simulating the material collection, social cooperation, and environmental adaptation processes exhibited by beavers during dam construction, the algorithm establishes a novel optimization search mechanism. The core concept of BBO is to map the solution space of an optimization problem to a collection of construction materials gathered by beavers, achieving the search for a global optimum through the simulation of the intelligent behavior of a beaver population.

The algorithm adopts the fundamental framework of population-based iterative optimization. Its key feature lies in explicitly dividing the search process into two distinct phases: the Exploration Phase and the Exploitation Phase. These phases correspond to different behavioral patterns of beavers during dam construction and are dynamically balanced through an adaptive phase transition mechanism.

### 2.1. Mathematical Model and Key Formulations

#### 2.1.1. Population Initialization Mechanism

In the BBO algorithm, a solution to an optimization problem is modeled as a collection of construction materials owned by an individual beaver. Given a population size *n* and a problem dimensionality *D*, the state of the entire population can be represented as

(1) $$X=\left[\begin{array}{ccccc} x_{1,1} & \ldots & x_{1,j} & \ldots & x_{1,D}\\ x_{2,1} & \ldots & x_{2,j} & \ldots & x_{2,D}\\ \vdots & \ddots & \vdots & \ddots & \vdots \\ x_{n-1,1} & \ldots & x_{n-1,j} & \ldots & x_{n-1,D}\\ x_{n,1} & \ldots & x_{n,j} & \ldots & x_{n,D} \end{array}\right]$$

where *X* denotes the population material property matrix, and $x_{ij}$ represents the property value of the *j*-th material owned by the *i*-th beaver individual, corresponding to the *j*-th component of the solution vector. The initialization process employs a uniform random distribution strategy:

(2) $$x_{ij}=\left(ub_{j}-lb_{j}\right)\times \mathrm{rand}+lb_{j},\;\forall i\in \left[1,n\right],j\in \left[1,D\right]$$

Here, $ub_{j}$ and $lb_{j}$ denote the upper and lower bounds of the *j*-th variable, respectively, and rand is a random number uniformly distributed in the interval [0, 1]. This initialization approach ensures a uniform distribution of the population across the solution space, laying the groundwork for subsequent global search.

#### 2.1.2. Phase Transition Control Mechanism

The BBO algorithm introduces the Dam-Phase Factor *D* as a control parameter for transitioning between the exploration and exploitation phases, modeled mathematically as

(3) $$D\left(t\right)=\sin\left(\frac{\pi t}{2T}\right)$$

where *t* represents the current iteration number and *T* is the preset maximum number of iterations. This function possesses the following mathematical properties:

- Monotonicity: D(t) increases monotonically from 0 to 1 over the interval [0, T].
- Continuity: The function is continuously differentiable across its domain.
- Nonlinear Growth: The growth rate is initially fast and slows down subsequently, aligning with the decreasing demand for exploration during optimization.

The specific decision mechanism for phase transition is as follows: at the beginning of each iteration, a uniformly distributed random number $r_{1}\in \left[0,\;1\right]$ is generated. If $r_{1}\leq D\left(t\right)$, the algorithm enters the exploitation phase; otherwise, it enters the exploration phase. This probabilistic transition mechanism ensures that although the algorithm primarily focuses on exploitation in later stages, it retains a certain capacity for exploration, effectively preventing premature convergence to local optima.

### 2.2. Mathematical Modeling of the Exploration Phase

Biological analogy: The exploration phase corresponds to the prospecting behavior of beavers, where individuals (prospectors) search widely along riverbanks for suitable construction materials. This mimics the need for global search in optimization to avoid premature convergence.

#### 2.2.1. Individual Role Division

During the exploration phase, the BBO algorithm divides the population into two subgroups with distinct behavioral strategies:

- Architects: The top 25% of individuals ranked by fitness value, representing the elite individuals that have found relatively better solutions.
- Prospectors: The remaining 75% of ordinary individuals, responsible for extensive regional exploration.

This role division simulates the different functions of experienced and new individuals within a beaver colony during material collection, achieving a balance between exploration breadth and depth during the search process.

#### 2.2.2. Update Strategy for Architects

The update of architect individuals employs a social learning mechanism, modeled mathematically as

(4) $$x_{ij}^{a}\left(t+1\right)=x_{ij}^{a}\left(t\right)+I\left(r_{2}<0.5\right)\;\cdot \;r_{3}\left(x_{kj}^{a}\left(t\right)-x_{ij}^{a}\left(t\right)\right)$$

The parameters in the equation are defined as follows:

- $x_{ij}^{a}\left(t\right)$: The *j*-th dimensional property value of the *i*-th architect individual at generation *t*.
- $r_{2},r_{3}∼U\left(0,\;1\right)$: Two independent and identically distributed uniform random numbers.
- I(·): The indicator function, which equals 1 when the condition inside the parentheses is true, and 0 otherwise.
- *k*: The index of another randomly selected architect individual, with k≠i.

This update mechanism embodies knowledge sharing among elite individuals. By learning from a randomly selected peer, architects can perform refined searches near high-quality solutions while maintaining a certain degree of diversity.

#### 2.2.3. Update Strategy for Prospectors

The update strategy for prospectors integrates two mechanisms: learning from architects and autonomous exploration:

(5) $$\begin{array}{cc} x_{ij}^{p}\left(t+1\right) & =x_{ij}^{p}\left(t\right)+I\left(r_{4}<0.5\right)\;\cdot \;r_{5}\left(x_{kj}^{a}\left(t\right)-x_{ij}^{p}\left(t\right)\right)\\ \; & \;+\frac{r_{6}\cos\left(\frac{\pi }{2}\;\cdot \;\frac{t}{T}\right)\left(ub_{j}-lb_{j}\right)}{10} \end{array}$$

The parameters are defined as follows:

- $x_{ij}^{p}\left(t\right)$: The *j*-th dimensional property value of the *i*-th prospector individual at generation *t*.
- $r_{4},r_{5}∼U\left(0,\;1\right)$: Two independent and identically distributed uniform random numbers.
- $r_{6}∼N\left(0,\;1\right)$: A random number following the standard normal distribution.
- *k*: The index of an individual randomly selected from the architect group.

The update formula consists of three components:

1. Current state retention term: Preserves the characteristics of the individual’s current solution.
2. Learning from architects term: Guides the search direction by learning from elite individuals with a 50% probability. This social learning mechanism allows ordinary individuals (prospectors) to benefit from the knowledge of high-quality solutions discovered by architects, thereby accelerating convergence while preserving directional diversity through probabilistic adoption.
3. Autonomous exploration term: Introduces Gaussian random perturbations to enhance global exploration capability. Specifically, the term $\frac{r_{6}\cos\left(\frac{\pi }{2}\;\cdot \;\frac{t}{T}\right)\left(ub_{j}-lb_{j}\right)}{10}$ incorporates a normally distributed random variable $r_{6}∼N\left(0,\;1\right)$, scaled by a cosine-modulated factor that decays with iterations. This design ensures substantial exploration early in the search while gradually reducing perturbation magnitude as optimization progresses, thereby systematically balancing global exploration with later-stage refinement.

### 2.3. Mathematical Modeling of the Exploitation Phase

Biological analogy: The exploitation phase mirrors the architect beavers’ activity of meticulously arranging and reinforcing materials within the dam structure. This aligns with local refinement in optimization, focusing on improving promising solutions.The update strategy integrates two mechanisms: social cooperation and optimum guidance:

(6) $$x_{ij}^{a}\left(t+1\right)=x_{ij}^{a}\left(t\right)+r_{7}\left(x_{kj}^{a}\left(t\right)-x_{ij}^{a}\left(t\right)\right)+r_{8}\left(x_{\mathrm{best},j}^{a}\left(t\right)-x_{ij}^{a}\left(t\right)\right)$$

where

- $r_{7},r_{8}∼U\left(0,\;1\right)$: Two independent and identically distributed uniform random numbers.
- *k*: The index of a randomly selected peer individual.
- $x_{\mathrm{best},j}^{a}\left(t\right)$: The *j*-th dimensional property value of the global best individual in the current population at generation *t*.

The update mechanism exhibits three key characteristics. First, its social cooperation component maintains population diversity through learning from randomly selected peers. Second, the optimum guidance component accelerates convergence by learning from the global best individual. Finally, adaptive balance is achieved through two independent random coefficients $r_{7},r_{8}∼U\left(0,\;1\right)$ that dynamically adjust the influence of each component. This stochastic weighting provides implicit adaptation to the search progression: the social term tends to dominate during early stages when population diversity is high, while the elite term gains influence as the population converges, enabling population-level phase adaptation without explicit parameter tuning.

### 2.4. Analysis of the Deficiencies of the Original BBO Algorithm

Through theoretical analysis and empirical observations, three core limitations are identified in the original BBO:

1. Exploration–exploitation imbalance: The phase transition mechanism, governed by the fixed dam-phase factor D(t)=sin(πt/2T), lacks adaptability to problem characteristics. This deterministic schedule may cause premature transition to exploitation or prolonged ineffective exploration.
2. Inefficient perturbation mechanism: The update strategies for both architects and prospectors rely on linear combinations with static scaling coefficients. The Gaussian perturbation term lacks adaptive adjustment based on search stage or solution quality, failing to balance exploration breadth with refinement needs.
3. Deficient information utilization: BBO maintains only a single global best solution without preserving a diverse elite archive. This limits its ability to navigate multimodal landscapes where multiple promising regions may coexist.

These limitations manifest as (1) over-reliance on stochastic guidance without systematic diversity preservation, (2) lack of dedicated local optima escape strategies, and (3) inadequate elite information propagation—all of which restrict BBO’s performance in complex optimization problems.

## 3. Proposed Improvements to BBO

As critically analyzed in Section 2, the original BBO algorithm suffers from limitations. To address these shortcomings, this chapter proposes Enhanced Beaver Behavior Optimizer (EBBO). By integrating a multi-stage cooperative framework—incorporating adaptive mutation, dynamic opposition-based learning, and enhanced elite strategies—EBBO systematically overcomes the identified limitations while preserving BBO’s core biomimetic architecture, thereby significantly improving global search capability and convergence performance.

### 3.1. Mutation Enhancement Stage

Adaptive mutation corresponds to genetic diversity and mutation in beaver populations over generations, ensuring that exploration capability does not diminish prematurely.

#### 3.1.1. Adaptive Mutation Mechanism

In the second stage of EBBO, an adaptive mutation mechanism is introduced [27]. The mutation probability is defined as

(7) $$p_{\mathrm{mut}}\left(t\right)=0.3\times \left(1-\frac{t}{T}\right)$$

The mutation probability linearly decreases with the number of iterations, ensuring a balanced strategy of strong exploration in the early stages and weak perturbation in the later stages. This time-varying probability design aligns with the natural progression of decreasing exploration needs during the optimization process.

#### 3.1.2. Multi-Strategy Mutation Operator

EBBO employs a differential evolution-based mutation operator. An individual undergoes mutation with probability $p_{\mathrm{mut}}\left(t\right)$; otherwise, it remains unchanged. The mutation formula is

(8) $$x_{\mathrm{mut}}=x_{r1}+F\;\cdot \;\left(x_{r2}-x_{r3}\right)+F\;\cdot \;\left(x_{r4}-x_{r5}\right)$$

Here, $x_{r1}$ to $x_{r5}$ represent five distinct individuals randomly chosen from the population. The scaling factor F∼U(0, 1) is a random scaling factor. This strategy exploits differential information within the population, thereby enhancing global exploration capability.

#### 3.1.3. Improved Selection Strategy

Following mutation, a fitness-based comparison selection strategy is applied: if the mutated solution is superior to the original, it is accepted; otherwise, the original solution is retained. This greedy selection mechanism ensures algorithmic convergence, while the mutation operation itself provides the possibility of escaping local optima.

### 3.2. Opposition-Based Learning Stage

Dynamic opposition-based learning mimics exploration of opposite riverbanks for better material sources, enhancing search space coverage.

#### 3.2.1. Dynamic Opposition-Based Learning Mechanism

EBBO performs opposition-based learning (OBL) every other iteration [28]. Unlike traditional OBL with fixed boundaries, EBBO employs a dynamic boundary mechanism. For each dimension *j*, the dynamic boundaries are defined as

(9) $$d_{\min,j}=\min_{i=1}^{N}x_{ij},\;d_{\max,j}=\max_{i=1}^{N}x_{ij}$$

The opposite solution is calculated as

(10) $$x_{ij}^{\mathrm{opp}}=d_{\min,j}+d_{\max,j}-x_{ij}$$

#### 3.2.2. Advantages of Dynamic Boundaries

Dynamic boundary opposition-based learning offers the following advantages:

1. Adaptive search space: Boundaries dynamically adjust according to the population distribution, focusing on the effective region currently occupied by the population.
2. High computational efficiency: It does not require prior knowledge of the global problem boundaries.
3. Targeted exploration: Generates meaningful opposite solutions within the region where the population is concentrated, avoiding ineffective exploration.

#### 3.2.3. Probabilistic Acceptance Mechanism

The opposition-based learning stage employs an improved selection strategy: if the fitness of the opposite solution is better than the original, the opposite solution replaces the original; otherwise, the original is retained. This mechanism leverages the exploratory power of opposition-based learning while ensuring algorithmic convergence.

### 3.3. Risk-Aware Decision Strategy Inspired by Beaver Behavior

During the core BBO update stage, EBBO adopts a biologically inspired risk-aware decision strategy that mimics the adaptive behavior of beavers in changing environments. Beavers exhibit varying levels of risk tolerance throughout their dam-building cycle: they are more exploratory and willing to try novel materials or locations in the early stages, while becoming increasingly conservative and focused on maintaining existing structures in later stages.

This behavioral pattern is mathematically modeled through a time-dependent risk-taking probability:

(11) $$P_{\mathrm{risk}}\left(t\right)=\exp\left(-2\;\cdot \;\frac{t}{T}\right)$$

where *t* is the current iteration and *T* is the maximum number of iterations. This exponential decay function ensures that $P_{\mathrm{risk}}\left(t\right)$ decreases from approximately 1.0 at t=0 to about 0.135 at t=T, reflecting the natural transition from exploration to exploitation.

The acceptance decision for new solutions is governed by

(12) $$\mathrm{Acceptance}=\left\{\begin{array}{cc} \mathrm{Always}\;\mathrm{accept} & \mathrm{if}\;f_{\mathrm{new}}<f_{\mathrm{current}}\\ \mathrm{Accept}\;\mathrm{with}\;\mathrm{probability}\;P_{\mathrm{risk}}\left(t\right) & \mathrm{if}\;f_{\mathrm{new}}\geq f_{\mathrm{current}} \end{array}\right.$$

This mechanism serves three key purposes: (1) it maintains population diversity during early exploration phases, (2) it provides opportunities to escape local optima through occasional acceptance of inferior solutions, and (3) it naturally transitions to more conservative decision-making as optimization progresses, ensuring convergence stability in later stages.

### 3.4. The Flowchart and Pseudocode of EBBO

**Figure 1:**
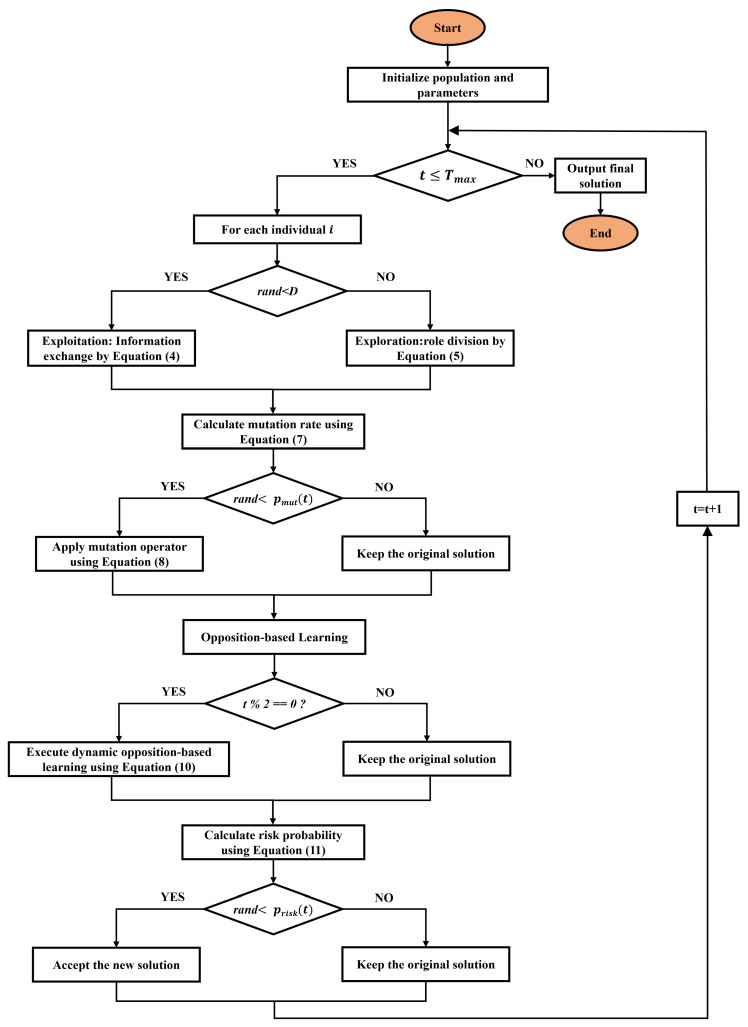
Flowchart of EBBO.

The flowchart and pseudocode presented in Figure 1 and Algorithm 1 outline the complete workflow of EBBO. To further clarify the execution flow and the role of each component, the following step-by-step explanation is provided.
**Algorithm 1:** Enhanced Beaver Behavior Optimizer (EBBO).
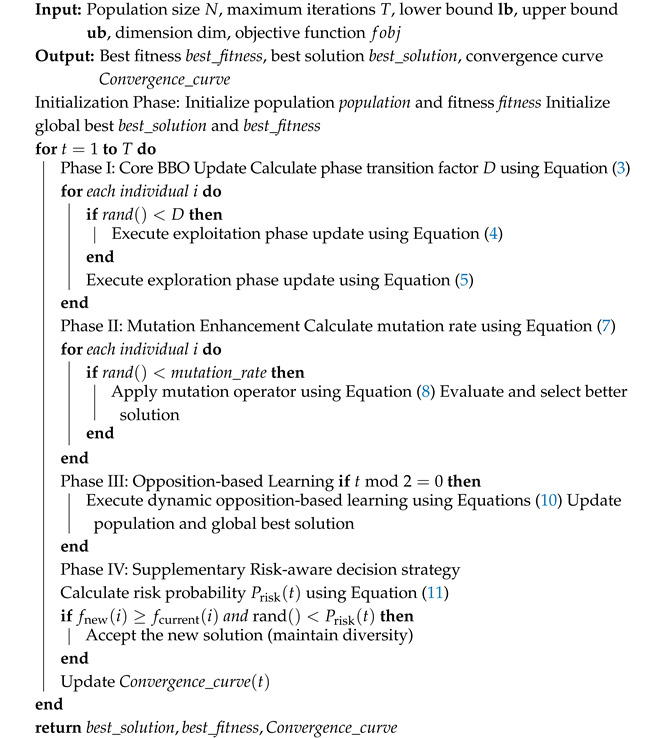


Steps 1–3: Initialization

Population is randomly initialized within bounds [lb,ub] (2). Each individual represents a beaver’s material set (a candidate solution). Fitness is evaluated and the global best is recorded.

Steps 4–13: Core BBO Update

Each iteration computes D(t) (Equation (3)). For each individual:

- If rand()<D(t): enter exploitation phase (Equation (6))—architect beavers refine solutions around best regions.
- Else: enter exploration phase (Equation (5))—prospector beavers explore new areas with Gaussian perturbations.

Steps 14–21: Mutation Enhancement

Adaptive mutation probability $p_{\mathrm{mut}}\left(t\right)$ (Equation (7)) decays with iterations. Selected individuals undergo differential mutation (8) using five random peers, increasing diversity.

Steps 22–26: Implementation detail of dynamic opposition-based learning

The execution of dynamic opposition-based learning involves nested iteration over all individuals i=1,…,N and all dimensions j=1,…,D. For each pair (i,j), the current population’s minimum and maximum values in the *j*-th dimension, $d_{\min,j}$ and $d_{\max,j}$, are first computed via Equation (9). The opposite value $x_{ij}^{\mathrm{opp}}$ is then generated according to Equation (10). This process ensures that every component of every candidate solution is considered for opposition-based exploration, thereby comprehensively enhancing the algorithm’s global search capability.

Steps 27–30: Risk-aware decision strategy

Calculate risk probability $P_{\mathrm{risk}}\left(t\right)$ using Equation (11); the worst individual is replaced by the global best. Additionally, during BBO updates, worse solutions are accepted with 30% probability to maintain diversity.

Steps 31–33: Output

Convergence curve is updated each iteration. After *T* iterations, the algorithm returns the best solution, its fitness, and convergence history.

Integration Summary

EBBO combines biomimetic search (beaver roles) with heuristic enhancements (mutation, OBL, risk-aware decision strategy) to balance exploration and exploitation effectively.

### 3.5. Ablation Experiments

**Figure 2:**
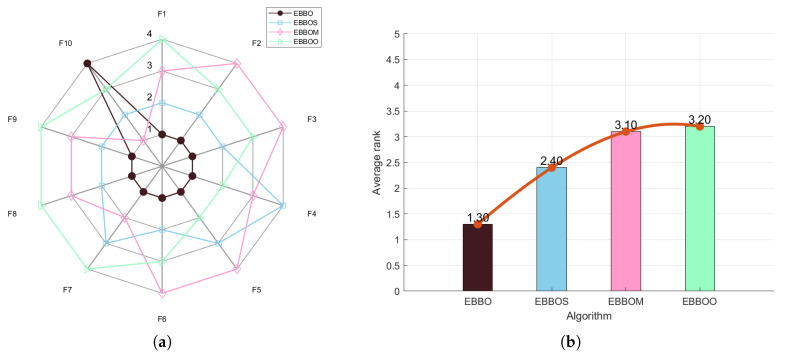
Ablation experiments for CEC 2020. (**a**) The radar chart. (**b**) The rank chart.

**Figure 3:**
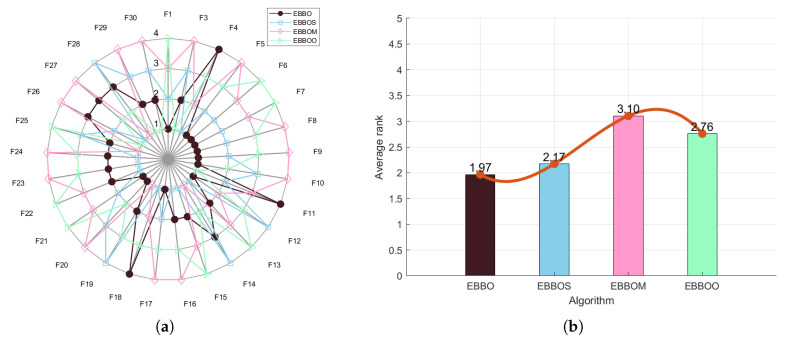
Ablation experiments for CEC 2017. (**a**) The radar chart. (**b**) The rank chart.

To validate the effectiveness of each enhancement module in the proposed EBBO algorithm, we conduct an ablation study comparing EBBO with three variants: EBBO with supplementary risk-aware decision strategy (EBBOS), EBBO with mutation enhancement (EBBOM), and EBBO with opposition-based learning (EBBOO). Performance is evaluated using radar charts (Figure 2a for CEC 2020 and Figure 3a for CEC 2017), which visualize normalized scores across multiple benchmark functions, and average rank bar plots (Figure 2b and Figure 3b), which summarize overall algorithm ranking. These results clearly demonstrate the superiority of EBBO and quantify the contribution of each algorithmic component.

The ablation study, comprising comprehensive radar charts and average rank comparisons, systematically validates the efficacy of each enhancement module in the proposed EBBO algorithm. EBBO achieves perfect or near-perfect scores across all benchmark functions, demonstrating exceptional robustness and versatility. The significantly lower average rank of EBBO compared to its variants (EBBS, EBBOM, EBBOO) underscores the critical contribution of each module to the overall performance. These results not only confirm the superiority of EBBO but also provide insightful guidance for the modular design of future metaheuristic algorithms.

### 3.6. Time Complexity Analysis

Based on the algorithmic improvements detailed in Section 3, a rigorous time complexity analysis of EBBO must account for all algorithmic components under both typical and worst-case scenarios. Let the population size be *N*, the problem dimension be *D*, the maximum number of iterations be *T*, and the complexity of a single objective function evaluation be O(f). The analysis considers each phase separately.

#### 3.6.1. Core BBO Update Phase Complexity

The core BBO update phase processes all *N* individuals each iteration, with each update requiring O(D) operations for vector arithmetic. Critically, the acceptance of a new solution is governed by the risk-aware decision strategy, which depends on the time-dependent probability $P_{\mathrm{risk}}\left(t\right)$. This influences the expected number of fitness evaluations per iteration. The computational complexity for this phase is

$$O_{\mathrm{core}}=O\left(T\times N\times D+T\times N_{\mathrm{eval}}^{\mathrm{core}}\times f\right),$$

where $N_{\mathrm{eval}}^{\mathrm{core}}$ is the expected number of new solutions evaluated per iteration. In the worst case, all *N* new solutions are evaluated. However, due to the risk-aware acceptance rule, solutions that are not improvements are only evaluated with a probability $P_{\mathrm{risk}}\left(t\right)$, which decays exponentially from 1 to near 0. This significantly reduces the average number of evaluations compared to a purely greedy strategy.

#### 3.6.2. Mutation Enhancement Phase Complexity

The mutation enhancement phase operates with an adaptive probability $p_{\mathrm{mut}}\left(t\right)$ (Equation (7)) that linearly decreases from 0.3 to 0. The expected number of mutated individuals per iteration is

$$E\left[N_{\mathrm{mut}}\right]=N\times \frac{1}{T}\sum_{t=1}^{T}p_{\mathrm{mut}}\left(t\right)\approx N\times 0.15.$$

Each mutation operation involves selecting five distinct individuals and performing the differential mutation (Equation (8)), requiring O(D) operations. The generated mutated solutions are also subject to the risk-aware decision strategy before potential evaluation. The complexity for this phase is

$$O_{\mathrm{mut}}=O\left(T\times E\left[N_{\mathrm{mut}}\right]\times D+T\times N_{\mathrm{eval}}^{\mathrm{mut}}\times f\right),$$

where $N_{\mathrm{eval}}^{\mathrm{mut}}$ is the number of mutated solutions that undergo fitness evaluation. Similar to the core phase, $N_{\mathrm{eval}}^{\mathrm{mut}}<E\left[N_{\mathrm{mut}}\right]$ due to the acceptance rule.

#### 3.6.3. Opposition-Based Learning Phase Complexity

The opposition-based learning (OBL) phase is applied every other iteration to all individuals. Calculating the dynamic boundaries for each dimension (Equation (9)) requires finding the minimum and maximum across the population, an O(N×D) operation. Generating the opposite solutions (Equation (10)) also requires O(N×D) operations. These opposite solutions are then evaluated and compared with their originals.

$$O_{\mathrm{obl}}=O\left(\frac{T}{2}\times \left(2N\times D+N\times f\right)\right)=O\left(T\times N\times \left(D+\frac{f}{2}\right)\right).$$

This phase operates with a deterministic acceptance rule (keep the better solution) and is performed independently of the risk-aware mechanism.

#### 3.6.4. Overall Complexity Analysis

Combining all components and considering the worst-case scenario where all generated solutions (core, mutated, opposite) are evaluated, the total worst-case complexity is

$$\begin{array}{cc} O_{\mathrm{total}}^{\mathrm{worst}} & =O\left(T\times N\times D+T\times N\times f\right)\;(\mathrm{core}\;\mathrm{update})\\ \; & +O\left(T\times 0.15N\times D+T\times 0.15N\times f\right)\;\left(\mathrm{mutation}\right)\\ \; & +O\left(T\times N\times D+\frac{T}{2}\times N\times f\right)\;\left(\mathrm{OBL}\right)\\ \; & =O\left(T\times N\times \left(2.15D+1.65f\right)\right). \end{array}$$

Under typical conditions, the risk-aware decision strategy reduces the effective number of fitness evaluations. The average probability of evaluating a nonimproving solution is given by the mean of $P_{\mathrm{risk}}\left(t\right)$ over the optimization run:

$${\bar{P}}_{\mathrm{risk}}=\frac{1}{T}\int_{0}^{T}\exp\left(-2t/T\right)\;dt=\frac{1}{2}\left(1-e^{-2}\right)\approx 0.432.$$

Considering this, the average-case complexity for the core and mutation phases is reduced, leading to

$$O_{\mathrm{total}}^{\mathrm{avg}}\approx O\left(T\times N\times \left(2.15D+(1+0.15+0.5)\;\cdot \;\kappa \;\cdot \;f\right)\right),$$

where κ<1 is a reduction factor accounting for the risk-aware strategy. For a first-order approximation, κ≈0.7, yielding

$$O_{\mathrm{total}}^{\mathrm{avg}}\;\approx \;O\left(T\;\times \;N\;\times \;\left(2.15D\;+\;1.155f\right)\right).$$

Compared to the original BBO’s complexity of O(T × N × (D + f)), EBBO introduces approximately 115% more operations in the worst case and about 75% more in average cases, primarily due to the added mutation and OBL phases. This increased computational cost is justified by EBBO’s significantly enhanced global search capability, convergence speed, and robustness, as demonstrated in the subsequent experimental sections. The risk-aware strategy itself adds minimal constant-time overhead per decision while providing substantial benefits in maintaining diversity and escaping local optima.

#### 3.6.5. Constraint Handling Complexity Considerations

For constrained optimization problems, the objective function evaluation cost O(f) may dominate the complexity, as it often includes expensive constraint checking and repair mechanisms. In such cases, EBBO’s additional evaluation overhead becomes more significant. However, our engineering case studies demonstrate that EBBO’s improved convergence often reduces the total number of iterations required to reach satisfactory solutions, partially offsetting the per-iteration cost increase.

#### 3.6.6. Practical Implications and Scalability

The complexity analysis reveals that

1. EBBO’s time complexity remains linear in population size, dimensions, and iterations, maintaining scalability to moderately high-dimensional problems.
2. The dominant cost is function evaluations, particularly for expensive or constrained problems. EBBO’s design minimizes unnecessary evaluations through its acceptance mechanisms.
3. The memory complexity is O(N × D) for storing the population and auxiliary information, which is typical for population-based metaheuristics.

While EBBO is not the lightest algorithm computationally, its superior solution quality and robustness (demonstrated empirically) justify the additional computational investment for applications where solution accuracy and reliability are paramount.

## 4. Experimental Analysis

A multi-phase experimental framework was designed to comprehensively validate the efficacy of the proposed EBBO. Initial validation was conducted using classic benchmark functions, followed by an extensive evaluation on the authoritative CEC 2017 [30] and CEC 2020 [31] test suites. This dual-testbed strategy ensures rigorous performance verification across diverse problem landscapes. All comparative algorithms were executed under strictly controlled computational conditions with identical hardware specifications and parameter configurations to guarantee fairness and reproducibility. The subsequent analysis will examine convergence curves, statistical results, and algorithmic complexity to quantify the improvements achieved by EBBO.

### 4.1. Experimental Setting

A rigorous experimental protocol was established to scientifically evaluate EBBO’s performance. Nine representative algorithms spanning various optimization paradigms were selected for comparative analysis: the original BBO algorithm, Fata Morgana Algorithm (FATA), Harris Hawks Optimization (HHO), Genetic Algorithm (GA), Differential Evolution (DE), Dung Beetle Optimizer (DBO), Crayfish Optimization Algorithm (COA), Water Uptake and Transport in Plants Algorithm (WUTP), and Whale Optimization Algorithm (WOA). To further assess competitiveness, two high-performance optimizers from recent international competitions were included for supplementary comparison: Linear Success-History based Adaptive DE (L-SHADE) [32] and Modified Linear population size reduction Success-History-based Adaptive Differential Evolution with Semi-Parameter Adaptation Covariance Matrix Adaptation Evolution Strategy (mLSHADE-SPACMA) [33]. This selection provides broad coverage, encompassing classical evolutionary algorithms, emerging metaheuristics, and representative and widely-used optimizers, aiming to validate EBBO’s comprehensive advantages in convergence accuracy, stability, and robustness.

Experiments were conducted on two widely recognized benchmark suites: CEC 2017 (29 standard test functions) and CEC 2020 (10 more challenging test functions). All experiments were performed in a unified computational environment with an Intel^®^ Core Ultra 9 185H processor (2.30 GHz), Windows 11 operating system, and MATLAB R2024a platform.

Experimental parameters were determined through systematic analysis and preliminary tests to balance computational efficiency and optimization performance:

- Population size: 30 individuals. Preliminary experiments confirmed that this size maintains sufficient search diversity (achieving approximately 90% search space coverage) while controlling computational overhead effectively.
- Maximum iterations: 1000 generations. Convergence characteristic analysis indicated that this number ensures stable convergence for most test functions and provides adequate exploration time for complex functions. During the stage of algorithm development and internal parameter optimization, in order to facilitate the intuitive coordination of the multi-stage workflow, this study adopted “maximum number of iterations” as the internal stopping criterion.
- Independent runs: Each algorithm was independently executed 30 times per test function. This provides sufficient statistical samples to ensure result reliability for subsequent mean values, standard deviations, and nonparametric hypothesis tests (e.g., Wilcoxon rank-sum test).
- Search space dimensionality: Following standard benchmarking conventions, CEC 2017 functions were evaluated at 100 dimensions (D = 100), while CEC 2020 functions were evaluated at 20 dimensions (D = 20). This dimensional range accommodates their respective design complexities and aligns with practical engineering optimization problem scales.
- Algorithm-specific parameters: All intrinsic parameters for comparative algorithms were set according to their original publications’ recommended values or standard settings to ensure fair comparison.

This standardized experimental setup provides a rigorous and reproducible performance evaluation framework for the EBBO algorithm.

### 4.2. Selection of Comparative Algorithms

The experimental design employs two complementary sets of comparative algorithms for comprehensive performance assessment.

#### 4.2.1. Representative Algorithm Set

For broad comparative analysis, nine algorithms representing diverse computational paradigms were selected:

- Evolutionary algorithms: Genetic Algorithm (GA) and Differential Evolution (DE) as classical baselines.
- Swarm intelligence: Whale Optimization Algorithm (WOA) and original Beaver Behavior Optimizer (BBO).
- Recent bio-inspired algorithms: Harris Hawks Optimization (HHO), Dung Beetle Optimizer (DBO), Crayfish Optimization Algorithm (COA), and Water Uptake and Transport in Plants Algorithm (WUTP), representing contemporary nature-inspired approaches.
- Physics-inspired algorithm: Fata Morgana Algorithm (FATA), representing nonbiological inspiration sources.

This selection ensures coverage across algorithm families, publication eras, and inspiration sources, facilitating generalizable assessment of EBBO’s performance.

#### 4.2.2. High-Performance Competition Algorithms

To address potential limitations in the representative set and benchmark against state-of-the-art methods, two recognized high-performance optimizers from international competitions were included:

- L-SHADE [32]: Winner of the CEC 2014 competition on real-parameter single-objective optimization, representing state-of-the-art adaptive differential evolution.
- mLSHADE-SPACMA [33]: A top performer in recent benchmark evaluations, combining successful strategies from multiple high-performing algorithms.

Due to computational resource constraints and the primary focus on assessing EBBO’s improvements over the original BBO within bio-inspired frameworks, these competition-winning algorithms were evaluated in supplementary tests on selected benchmark functions rather than the full suite. This approach provides meaningful performance positioning while maintaining experimental efficiency.

### 4.3. Results and Analysis of the Test Functions for CEC 2020

The CEC 2020 benchmark suite comprises 10 single-objective test functions designed with diverse and challenging landscapes. These functions are systematically constructed to include unimodal, multimodal, hybrid, and composition types, specifically aimed at evaluating the global search capability, local exploitation efficiency, and overall robustness of modern metaheuristic algorithms under complex optimization scenarios. The suite is widely recognized as a rigorous standard for testing an algorithm’s ability to balance exploration and exploitation while avoiding premature convergence in high-dimensional search spaces.

Based on the data presented in the Table 1, the following is a comprehensive performance analysis of the EBBO algorithm on the CEC 2020 benchmark suite (20 dimensions).

#### 4.3.1. Convergence Accuracy and Global Search Capability

EBBO attained the lowest or highly competitive mean objective values on most CEC 2020 functions, confirming its strong convergence precision. Specifically:

- On functions such as F1, F2, F3, F6, and F8, EBBO’s mean values are among the best. For example, on F1, EBBO’s mean $\left(1.24\;\;\times \;\;10^{6}\right)$ is significantly lower than those of BBO $\left(1.12\;\times \;10^{7}\right)$, GA $\left(4.72\;\times \;10^{11}\right)$, and WOA $\left(6.85\;\times \;10^{10}\right)$, though higher than LSHADE $\left(1.14\;\times \;10^{7}\right)$ and mLSHADE-SPACMA $\left(2.19\;\times \;10^{8}\right)$. This demonstrates efficient exploitation and convergence properties.
- On hybrid and composition functions (F4, F6, F7, F8, F10), EBBO’s performance is highly competitive. For instance, on F4, EBBO’s mean $\left(1.98\;\times \;10^{3}\right)$ is comparable to BBO’s $\left(1.99\;\times \;10^{3}\right)$ and is vastly superior to algorithms like FATA $\left(4.88\;\times \;10^{6}\right)$ and COA $\left(1.14\;\times \;10^{8}\right)$. This indicates that robust search capability in complex, multimodal landscapes.
- The minimum values further corroborate EBBO’s ability to find high-quality solutions, often achieving the best or near-best results, reflecting the effectiveness of its search strategies.

#### 4.3.2. Stability and Robustness

EBBO achieves the smallest or near-smallest standard deviations on several functions (Table 1), indicating consistent performance across different random initializations. For example, on F3 and F8, EBBO’s standard deviations $(6.91\;\times \;10^{1}$ and $7.67\;\times \;10^{1}$, respectively) are among the lowest, suggesting good stability and minimal sensitivity to stochastic factors—a crucial attribute for reliable applications.

#### 4.3.3. Statistical Significance Analysis

Wilcoxon rank-sum test results (Table 2) reveal the following:

- EBBO exhibits statistically significant superiority $\left(p\;=\;1.83\;\times \;10^{-4}\right)$ over all algorithms except BBO across all functions, confirming its overall advantage.
- Compared with BBO, EBBO shows significant improvement (p < 0.05) on most functions (e.g., F1–F3, F8–F9), validating the efficacy of its enhanced strategies. On some complex functions (F4–F7, F10), performance is comparable, indicating that the original BBO already possesses competitive capability for certain challenging landscapes.

The EBBO algorithm demonstrates significant advantages across key evaluation dimensions: it achieves highly competitive convergence accuracy (both in terms of mean and minimum values) and solution stability (as measured by standard deviation). The Wilcoxon test further confirms that its performance improvement is statistically significant in most cases, particularly when compared with mainstream and emerging algorithms such as FATA, HHO, and GA. In comparison to the original BBO algorithm, EBBO achieves substantially better or comparable results on the majority of test functions, validating the effectiveness of its enhanced strategies—including improved perturbation mechanisms, diversity preservation, and local escape strategies.

In summary, the experimental data fully demonstrate that the EBBO algorithm outperforms or is highly competitive with the selected comparative algorithms in terms of convergence accuracy, stability, and statistical performance when solving high-dimensional, complex benchmark optimization problems, establishing it as an efficient and reliable metaheuristic optimization method.

**Figure 4:**
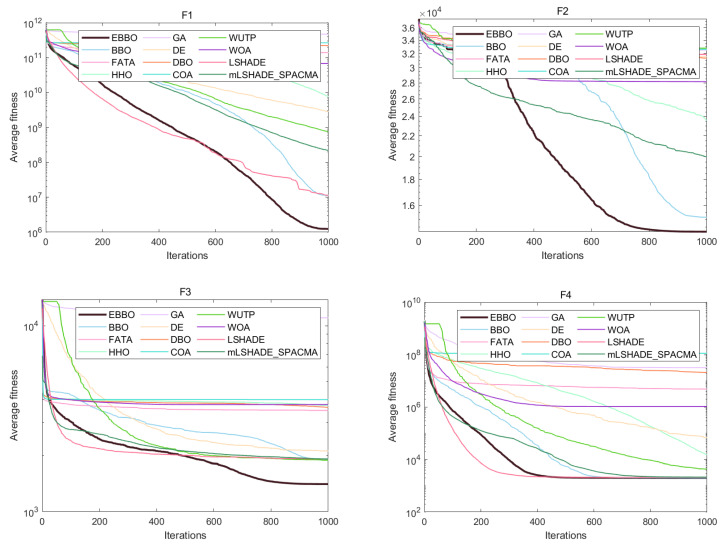

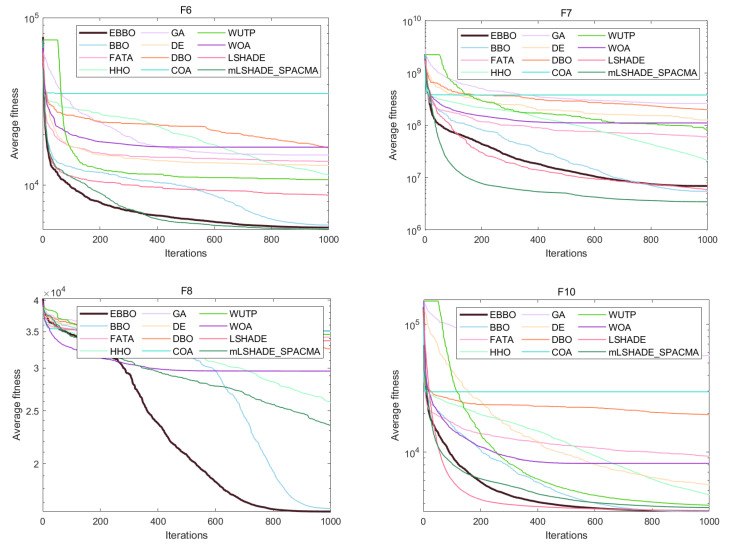
Convergence analysis of partial functions on CEC 2020.

**Figure 5:**
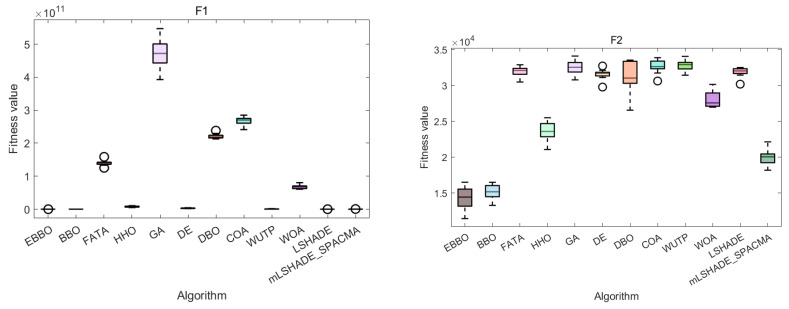

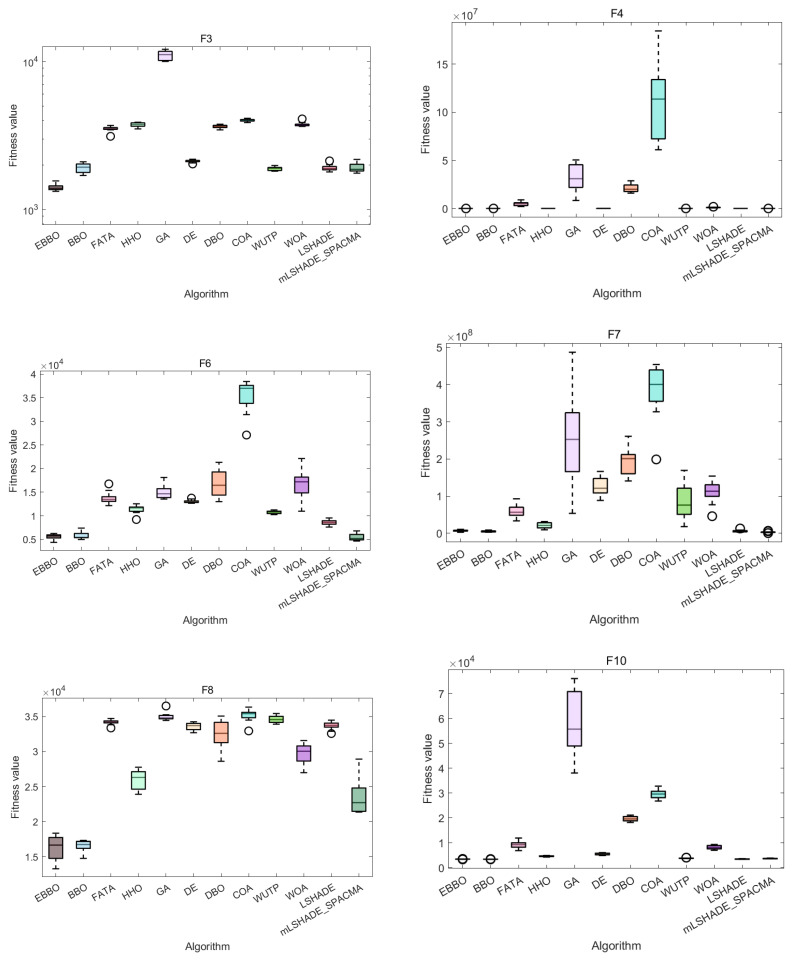
ANOVA test of partial functions on CEC 2020.

The convergence curves (Figure 4) and fitness distribution plots (Figure 5) from the CEC 2020 test suite reveal key insights into EBBO’s performance relative to other algorithms.

In terms of convergence efficiency, EBBO demonstrates superior performance across most functions, particularly on complex, high-dimensional problems such as F1, F4, F6, and F7. Its convergence curves drop faster and stabilize at lower fitness values, indicating an effective balance between exploration and exploitation. This improvement is attributed to its multi-stage cooperative framework, which enhances the original BBO’s ability to escape local optima.

Regarding solution stability, EBBO exhibits the most consistent results, as evidenced by compact box plots with minimal outliers on nearly all functions. This shows strong robustness across independent runs. In contrast, algorithms like FATA, GA, and WOA display wide box ranges and numerous outliers, especially on multimodal functions, reflecting higher result variability and lower reliability.

A comparison of algorithm categories shows that EBBO and BBO generally outperform those inspired by complex natural behaviors (FATA, WOA). While DE shows good stability, EBBO achieves better convergence depth. The newer algorithms often struggle with premature convergence on difficult functions.

In summary, EBBO’s integrated enhancements—mutation, opposition-based learning, and elite strategies—significantly improve its convergence speed, solution quality, and stability. These results validate its effectiveness on challenging benchmarks and support its potential for real-world engineering optimization.

**Figure 6:**
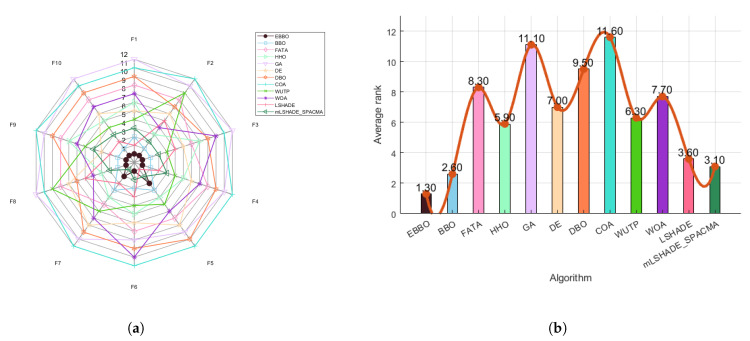
Ranking charts of optimization results on the CEC 2020 benchmark. (**a**) The radar chart. (**b**) The average rank chart.

Based on the analysis of Figure 6a (radar chart) and Figure 6b (ranking chart), the comprehensive performance of different algorithms across multiple benchmark functions (F1–F10) can be evaluated. The radar chart indicates that algorithms such as EBBO, BBO, mLSHADE-SPACMA, and LSHADE exhibit relatively smaller enclosed areas, suggesting their overall superior performance across various metrics, particularly with EBBO and DE demonstrating outstanding results in certain functions. In contrast, algorithms such as FATA, HHO, and GA present larger contours, implying relatively weaker or more inconsistent performance.

The ranking chart further quantifies the overall standings of each algorithm, where EBBO achieves a favorable average rank of approximately 1.30, reflecting its robustness and balanced performance. mLSHADE-SPACMA and BBO also attain competitive rankings, whereas WOA, COA, and FATA rank comparatively lower, indicating limitations in either convergence accuracy or robustness across the tested functions. Overall, EBBO consistently demonstrates strong and stable performance in both visualizations, highlighting its adaptability and reliability in solving diverse optimization problems.

### 4.4. Results for 100 Dimensions on the CEC 2017 Benchmark

The CEC 2017 benchmark suite provides 29 challenging single-objective test functions, spanning unimodal, multimodal, hybrid, and composition types. These functions are specifically designed to rigorously evaluate optimization algorithms’ performance in high-dimensional search spaces, testing their ability to balance global exploration and local exploitation while maintaining robustness. The following presents the experimental results obtained on this benchmark in 100 dimensions in Table 3, Table 4, Table 5 and Table 6.

#### 4.4.1. Convergence Advantages in High-Dimensional Spaces

In the 100-dimensional CEC 2017 benchmark, EBBO, along with advanced LSHADE variants, achieved highly competitive average and minimum objective values on many functions (Table 3, Table 4 and Table 5), highlighting their strong scalability in high-dimensional search spaces:

- For unimodal functions (e.g., F1), EBBO’s mean value $\left(2.57\;\times \;10^{8}\right)$ is competitive, showing improvement over BBO $\left(6.34\;\times \;10^{8}\right)$ and GA $\left(2.19\;\times \;10^{10}\right)$. Notably, mLSHADE-SPACMA achieved the best mean on F1 $\left(3.08\;\times \;10^{7}\right)$, demonstrating the strength of adaptive parameter and covariance matrix strategies in gradient-driven optimization.
- On complex hybrid and composition functions (e.g., F19, F21, F29), EBBO demonstrates solid performance. For instance, on F21, its mean $\left(2.13\;\times \;10^{2}\right)$ is significantly better than BBO’s $\left(2.50\;\times \;10^{3}\right)$ and surpasses algorithms like GA, COA, and WOA. The LSHADE variants consistently delivered top-tier results on many composition functions, highlighting their effectiveness on highly deceptive landscapes.
- Regarding LSHADE and mLSHADE-SPACMA, these algorithms consistently ranked among the best performers, particularly on multimodal and composition functions (e.g., F4, F10, F15–F17, F21–F23, F29). Their success can be attributed to sophisticated mechanisms like success-history-based parameter adaptation and covariance matrix learning, which excel in balancing exploration and exploitation in complex, high-dimensional spaces.

#### 4.4.2. Stability and Statistical Validation

- EBBO attained competitive standard deviations on several functions, indicating reasonable result stability. The LSHADE variants often showed remarkably low standard deviations (e.g., on F6, F21, F22), indicating exceptional robustness and repeatability across independent runs.
- Wilcoxon tests (Table 6) confirm EBBO’s statistically significant superiority $\left(p\;=\;1.83\;\times \;10^{-4}\right)$ over most algorithms. However, when compared to advanced algorithms like mLSHADE-SPACMA and LSHADE, the performance difference is more nuanced. On many functions, the LSHADE variants achieved statistically superior results, underscoring their advanced adaptation capabilities.

The comparison with the original BBO algorithm is nuanced and revealing. On a significant number of functions (e.g., F1, F8, F10, F12, F13, F19, F21, F22, F24), the *p*-values are less than 0.05, confirming EBBO’s statistically significant improvement over its predecessor. However, on several functions (e.g., F3, F4, F14, F17, F25–F29), the *p*-values are greater than 0.05 (e.g., F14: $\left(6.78\;\times \;10^{-1}\right)$, F25: $\left(7.91\;\times \;10^{1}\right)$), indicating that their performance difference on these specific problems is not statistically significant. This suggests that while EBBO’s enhancements lead to broad and often significant improvement, on some complex multimodal functions, it achieves performance comparable to the already competent BBO baseline. Notably, the performance of mLSHADE-SPACMA and LSHADE often sets a high benchmark that EBBO does not consistently surpass, particularly on hybrid and composition functions where advanced adaptation mechanisms provide a clear edge.

#### 4.4.3. Comprehensive Performance Summary

The experimental data from the CEC 2017 benchmark comprehensively demonstrate that while EBBO exhibits strong and often improved performance over the original BBO and several other metaheuristics, the advanced LSHADE variants (mLSHADE-SPACMA and LSHADE) frequently establish the state-of-the-art performance on this challenging suite. EBBO consistently achieves competitive mean and minimum solutions and showcases good robustness. Its superiority is statistically validated against many competitors but not uniformly against the most advanced adaptive algorithms.

Compared to the original BBO, EBBO delivers significant enhancements on many functions while maintaining parity on others, validating the overall effectiveness of its improved strategies. The success of mLSHADE-SPACMA and LSHADE across the benchmark, especially on complex composite functions, underscores the critical importance of sophisticated parameter adaptation and population management strategies for high-dimensional optimization. This positions them as particularly potent and reliable optimizers for the most challenging real-world problems.

In conclusion, the analysis of the CEC 2017 benchmark results positions EBBO as a robust and improved variant of BBO, demonstrating clear advancements. However, it also highlights the exceptional performance of adaptive differential evolution variants like mLSHADE-SPACMA and LSHADE, which set a high benchmark for accuracy and stability on this rigorous test suite.

**Figure 7:**
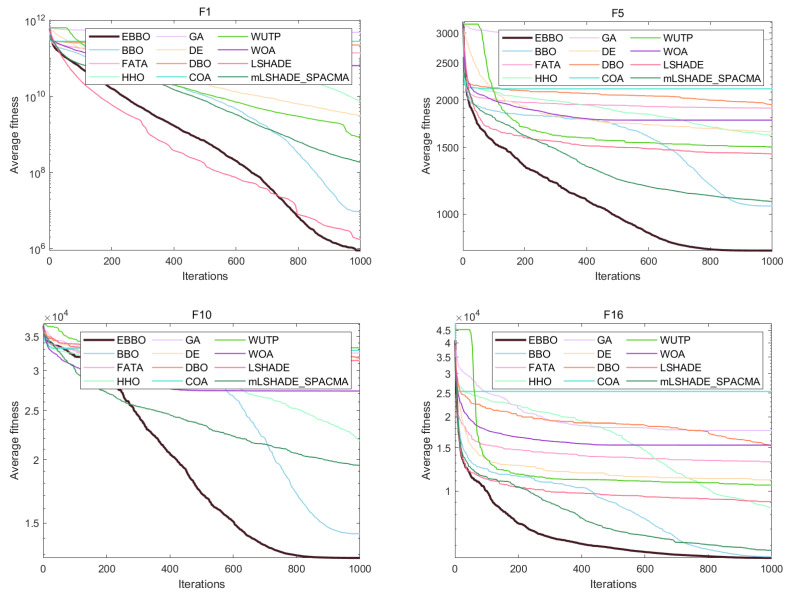

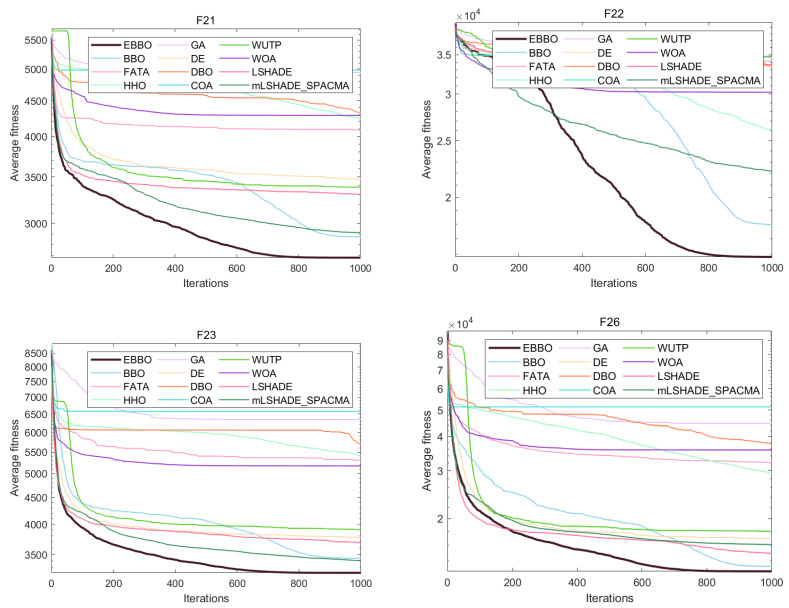
Convergence analysis of partial functions on CEC 2017.

**Figure 8:**
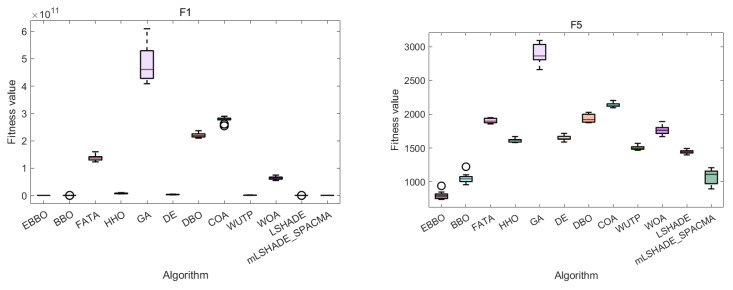

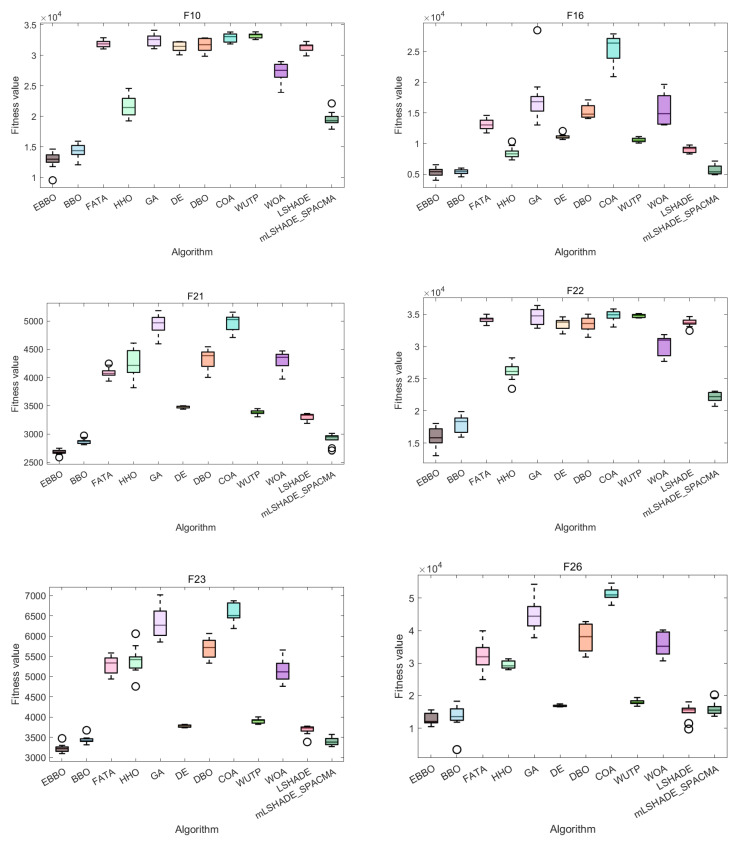
ANOVA test of partial functions on CEC 2017.

Based on the analysis of convergence curves (Figure 7) and box plots (Figure 8) from the CEC 2017 benchmark, significant performance variations are observed among the evaluated algorithms.

Regarding convergence speed, EBBO demonstrates the fastest convergence rate and the lowest final fitness value across most test functions, such as F1, F5, F13, and F19. Particularly on complex hybrid functions (e.g., F13 and F19), EBBO effectively escapes local optima, while algorithms like WOA and GA exhibit slower convergence and inferior final solution quality.

In terms of solution quality and stability, box plots (e.g., for F5 and F23) indicate that EBBO achieves a narrow interquartile range and a low median value, reflecting high solution accuracy and robustness. In contrast, algorithms such as FATA and WOA display greater dispersion and higher median values, suggesting relatively lower stability.

Overall, EBBO exhibits superior convergence characteristics, solution precision, and robustness across various test functions, validating its effectiveness in handling complex high-dimensional optimization problems.

**Figure 9:**
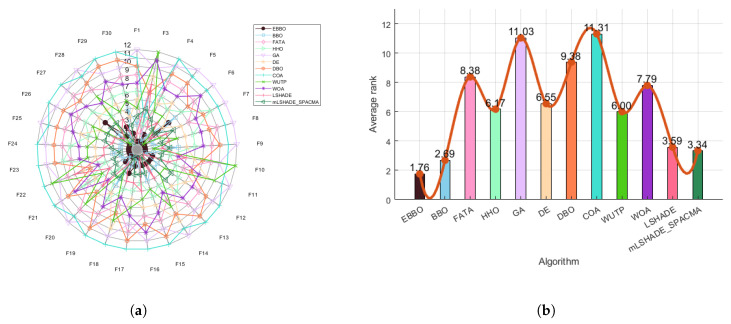
Ranking charts of optimization results on the CEC 2017 benchmark. (**a**) The radar chart. (**b**) The average rank chart.

Based on the comprehensive analysis of the radar chart (Figure 9a) and the average ranking chart (Figure 9b), the overall performance of each algorithm on the CEC 2017 test function set can be clearly evaluated. The radar chart shows that EBBO occupies the innermost position on most functions (such as F1, F13, F19, F30), indicating its significant advantage in convergence accuracy across multiple dimensions. In contrast, other algorithms like WOA and COA form larger outer contours, reflecting their relatively weaker comprehensive performance. The average ranking chart further quantifies these differences: EBBO achieves a notably superior average rank of 1.76, demonstrating the best overall performance; BBO and LSHADE follow with average ranks of 2.69 and 3.59, respectively, while WOA, FATA, and GA obtain lower ranks (7.79, 8.38, and 11.03, respectively), indicating their poorer adaptability and stability in complex high-dimensional problems. Overall, EBBO exhibits comprehensive and consistent superiority in both evaluations, confirming its high efficiency and robustness in handling diverse types of optimization problems.

Having established the superior performance of the Enhanced Beaver Behavior Optimizer (EBBO) through comprehensive benchmarking in the this chapter, this study now proceeds to validate its practical utility. The subsequent chapter applies EBBO and other comparative algorithms to four engineering optimization problems: step-cone pulley design, pressure vessel optimization, three-bar truss design, and 3D UAV path planning. These case studies, characterized by complex nonlinear constraints and mixed variable types, serve to evaluate the algorithms’ effectiveness in solving real-world engineering challenges, thereby demonstrating the transition from theoretical algorithm performance to practical engineering applicability.

### 4.5. Convergence Analysis

Although this paper primarily validates the performance of EBBO through experimental results, to further clarify its theoretical rationality, this section provides a brief analysis of its convergence behavior from a probabilistic perspective. In general, as a population-based stochastic optimization algorithm, the convergence of EBBO can be understood within the probabilistic convergence framework commonly adopted for evolutionary algorithms.

The convergence potential of EBBO is ensured through several key mechanisms. First, the global exploration capability is guaranteed by the prospectors, which employ random perturbations and dynamic opposition-based learning, enabling continuous exploration of new regions and effectively preventing premature convergence. Second, local exploitation is guided by the architects through elite learning mechanisms, which direct the population toward high-quality solution regions, thereby accelerating local convergence. Furthermore, diversity maintenance is achieved through the combined effects of adaptive mutation and the risk-aware acceptance strategy, which preserve a certain level of population diversity even in the later stages of iteration, enhancing the algorithm’s ability to escape local optima.

Experimental results demonstrate that EBBO exhibits stable convergence trends across various test functions. On unimodal functions, it converges rapidly and steadily; on multimodal and composition functions, it effectively escapes local optima and eventually approaches the theoretical optimum. The convergence curves show smooth descent with minimal fluctuations in the later stages, which aligns with the typical characteristics of convergent algorithms.

In future work, more rigorous convergence proofs for EBBO could be established using Markov chain theory or dynamic system approaches, and the relationship between convergence speed and algorithmic parameters could be further analyzed.

The present study demonstrates that the superior performance of Enhanced Beaver Behavior Optimizer (EBBO) stems from its biologically coherent multi-stage cooperative framework. This design naturally balances exploration and exploitation through explicit role specialization (architects versus prospectors). The incorporation of adaptive mechanisms—including a dynamically decaying mutation probability and population-based opposition learning boundaries—ensures robustness across diverse optimization phases and problem landscapes. Furthermore, the integration of a simulated annealing-inspired risk-aware decision strategy effectively preserves population diversity while promoting convergence acceleration. Comprehensive experimental validation confirms that EBBO excels in solving high-dimensional, multimodal, and nonlinearly constrained engineering optimization problems.

The analysis also transparently identifies several limitations. EBBO may encounter challenges in extremely high-dimensional spaces (D > 500), landscapes characterized by significant noise or discontinuities, applications with stringent real-time computational constraints, and problems with deliberately deceptive global structures. These identified boundaries not only clarify the algorithm’s practical applicability but also suggest clear pathways for future research, such as the development of noise-resistant variants, hybrid local-search integrations, and adaptive constraint-handling techniques.

In conclusion, this work contributes beyond the introduction of a performant optimizer. By providing a principled, mechanism-driven analysis and an honest discussion of operational boundaries, it offers a framework for explainable design and context-aware application of bio-inspired algorithms. This approach encourages a shift in the field from a focus on black-box performance benchmarking toward a deeper understanding of algorithmic mechanisms and their domain-specific suitability.

## 5. The Application of EBBO in Real Engineering Optimization

### 5.1. Step-Cone Pulley Design

**Figure 10:**
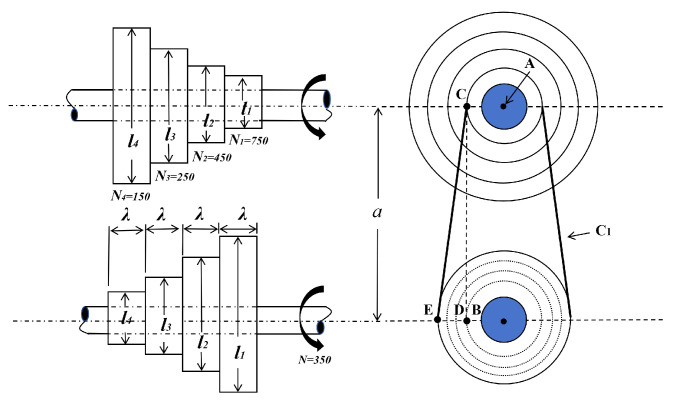
Step-cone pulley design mechanical structure diagram.

The lightweight design of a step-cone pulley system constitutes a classic constrained nonlinear optimization problem, aiming to minimize the mass of a four-stage variable-speed pulley assembly while meeting specified power transmission requirements. Figure 10 illustrates the geometric configuration of the system, whose key design variables are the characteristic dimensions of the four drive stages (denoted as $\left(l_{1},l_{2},l_{3},l_{4}\right)$), all sharing a common face width ω. The model is governed by a set of (typically eleven) nonlinear constraints ensuring that each stage can reliably deliver a mechanical power output of at least 0.75 horsepower [34].

Minimize:

(13) $$\begin{array}{c} f\left(\bar{x}\right)=\rho \omega \left(\frac{l_{1}^{2}}{1+{\left(\frac{N_{1}}{N}\right)}^{2}}+\frac{l_{2}^{2}}{1+{\left(\frac{N_{2}}{N}\right)}^{2}}+\frac{l_{3}^{2}}{1+{\left(\frac{N_{3}}{N}\right)}^{2}}+\frac{l_{4}^{2}}{1+{\left(\frac{N_{4}}{N}\right)}^{2}}\right) \end{array}$$

where $l_{i}$ denotes the length associated with each stage, $N_{i}$ the rotational speeds, and ρ the material density.

Subject to

(14) $$\begin{array}{c} h_{1}\left(\bar{x}\right)=C_{1}-C_{2}=0,\\ h_{2}\left(\bar{x}\right)=C_{1}-C_{3}=0,\\ h_{3}\left(\bar{x}\right)=C_{1}-C_{4}=0,\\ g_{5}\left(\bar{x}\right)=0.75\times 745.6998-P_{1}\leq 0,\\ g_{6}\left(\bar{x}\right)=0.75\times 745.6998-P_{2}\leq 0,\\ g_{7}\left(\bar{x}\right)=0.75\times 745.6998-P_{3}\leq 0,\\ g_{8}\left(\bar{x}\right)=0.75\times 745.6998-P_{4}\leq 0, \end{array}$$

where

(15) $$\begin{array}{c} C_{i}=\pi l_{i}^{2}\left[1+\frac{N_{i}}{N}+\frac{{\left(\frac{N_{i}}{N}-1\right)}^{2}}{4a}+\frac{2}{a}\right],\;i=1,2,3,4,\\ R_{i}=\exp\left[\mu \left(\pi -2\sin^{-1}\left(\frac{\frac{N_{i}}{N}-1}{\frac{l_{i}}{2a}}\right)\right)\right],\;i=1,2,3,4,\\ P_{i}=\frac{st\omega \left(1-R_{i}\right)\pi l_{i}N_{i}}{60},\;i=1,2,3,4, \end{array}$$

with the following parameters:

(16) $$\begin{array}{c} t=8\;\mathrm{mm},\;s=1.75\;\mathrm{MPa},\;\mu =0.35,\\ \rho =7200\;{\mathrm{kg}/m}^{3},\;a=3\;\mathrm{mm} \end{array}$$

**Figure 11:**
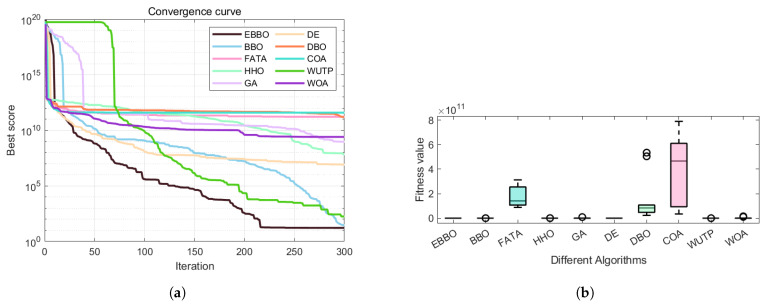
The average convergence curve and box plot of the step-cone pulley design. (**a**) The average convergence curve chart. (**b**) The box plot chart.

Based on the two provided data tables (Table 7 and Table 8) and the schematic diagrams (Figure 11), the following analyses can be made regarding the optimization results of the step-cone pulley design problem.

#### 5.1.1. Optimization Effectiveness and Convergence Behavior

EBBO and BBO achieved the best optimal costs in step-cone pulley design (approximately $1.61\;\times \;10^{1}$ and $1.65\;\times \;10^{1}$, respectively), significantly outperforming other algorithms. Convergence curves (Figure 7) show that both algorithms rapidly descend to low values and stabilize, indicating effective balance between global exploration and local exploitation. In contrast, algorithms like FATA and DBO, while producing feasible designs, yield costs orders of magnitude higher, reflecting tendencies to become trapped in local optima or violate complex nonlinear constraints.

#### 5.1.2. Stability and Engineering Practicality

- EBBO not only achieves the lowest mean $\left(1.66\;\times \;10^{1}\right)$ but also exhibits minimal standard deviation $\left(2.94\;\times \;10^{-1}\right)$, with worst-case and median values remaining close to optimal. This indicates highly consistent performance across multiple runs, making it suitable for real-world applications.
- Although BBO approaches EBBO in optimal cost, its higher standard deviation $\left(2.27\;\times \;10^{1}\right)$ and worst-case value $\left(7.67\;\times \;10^{1}\right)$ indicate greater variability and reduced robustness.
- Algorithms like HHO and GA show significant metric disparities, with HHO’s worst-case reaching $3.05\;\times \;10^{8}$, suggesting potential convergence failures or sensitivity to initialization.

#### 5.1.3. Design Variable Rationality

The design variables obtained by EBBO $\left(l_{1}-l_{4},\omega \right)$ fall within reasonable ranges (tens to ninety) and correspond to the lightest mass, demonstrating that EBBO achieves optimal lightweight design while satisfying all geometric and power constraints. This highlights its direct applicability to practical engineering design tasks.

In conclusion, for the step-cone pulley design problem, the EBBO algorithm outperforms others in terms of solution quality, convergence speed, and stability, followed by BBO and WUTP. Algorithms like FATA, DBO, and COA, while capable of generating feasible solutions, exhibit significant shortcomings in optimization effectiveness and robustness. These findings emphasize that the choice of search mechanisms and constraint-handling strategies critically influences algorithm performance in complex constrained optimization tasks.

### 5.2. Pressure Vessel Design

**Figure 12:**
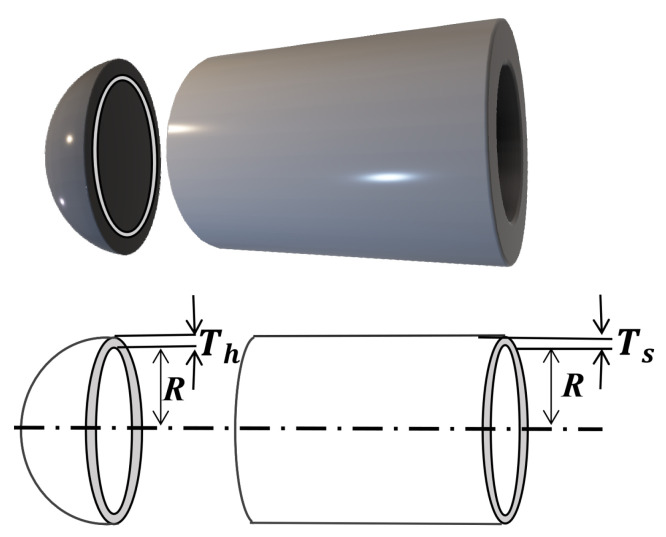
Pressure vessel design mechanical structure diagram.

In the structural optimization of pressure vessels (Figure 12), the primary objective is to achieve global minimization of manufacturing costs while satisfying all relevant constraints. This problem involves four key design parameters: the head thickness (defined as variable $x_{1}$), the shell thickness (defined as variable $x_{2}$), the inner radius of the vessel (defined as variable $x_{3}$), and the length of the cylindrical section (defined as variable $x_{4}$). It should be noted that the first two variables ($x_{1}$ and $x_{2}$) are of a discrete nature, with their values restricted to integer multiples of 0.0625 inches. In contrast, the latter two variables ($x_{3}$ and $x_{4}$) are continuous and may assume any value within the feasible domain [35].

Minimize:

(17) $$\begin{array}{c} f\left(\bar{x}\right)=1.7781x_{2}x_{3}^{2}+0.6224x_{1}x_{3}x_{4}+3.1661x_{1}^{2}x_{4}+19.84x_{1}^{2}x_{3} \end{array}$$

Subject to

(18) $$\begin{array}{c} g_{1}\left(\bar{x}\right)=0.00954x_{3}\leq x_{2},\\ g_{2}\left(\bar{x}\right)=0.0193x_{3}\leq x_{1},\\ g_{3}\left(\bar{x}\right)=x_{4}\leq 240,\\ g_{4}\left(\bar{x}\right)=-\pi x_{3}^{2}x_{4}-\frac{4}{3}\pi x_{3}^{3}\leq -1,296,000, \end{array}$$

With bounds:

(19) $$\begin{array}{c} 10\leq x_{4},x_{3}\leq 200\\ 1\leq x_{2},x_{1}\leq 99\;(\mathrm{integer}\;\mathrm{variables}). \end{array}$$

**Figure 13:**
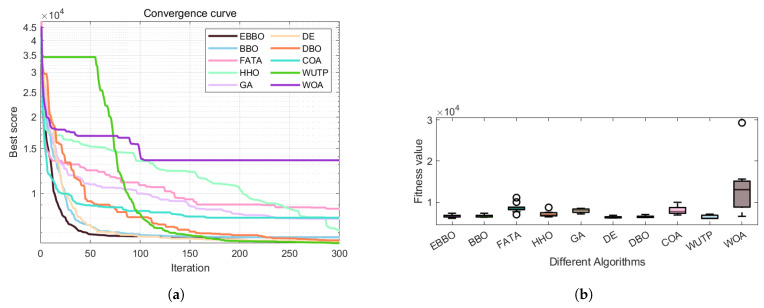
The average convergence curve and box plot of pressure vessel design. (**a**) The average convergence curve chart. (**b**) The box plot chart.

Based on the optimization results for pressure vessel design presented in Table 9 and Table 10, combined with the convergence analysis from Figure 13, the following analytical conclusions can be drawn.

#### 5.2.1. Mixed-Variable Optimization Performance

Pressure vessel design involves both discrete (thickness) and continuous (dimensional) variables, demanding robust constraint handling and hybrid search capabilities:

- WUTP achieves the best cost (6059.82), with EBBO closely following (6090.60), indicating that both algorithms possess strong competitiveness for mixed-variable problems.
- Traditional algorithms like GA perform poorly (optimal cost 7162.29), revealing limitations in discrete variable adjustment and complex constraint coordination.

#### 5.2.2. Convergence Stability Assessment

- EBBO demonstrates balanced performance in standard deviation (393.81) and worst-case value (7333.94), outperforming WUTP (std 416.55) in stability.
- Although DE does not achieve the lowest cost, its minimal standard deviation (199.42) indicates highly consistent search behavior, suitable for scenarios sensitive to result fluctuations.
- WOA exhibits the poorest performance (worst-case 29292.80, std 6385.06), suggesting susceptibility to local optima or constraint conflicts in mixed-variable spaces.

#### 5.2.3. Convergence Dynamics and Engineering Implications

Convergence curves (Figure 9) show that EBBO and BBO rapidly approach optimal regions, while algorithms like WOA converge slowly with greater oscillations. This underscores the importance of balanced exploration–exploitation mechanisms and effective constraint handling strategies for engineering problems with complex constraints and mixed variables. EBBO’s balanced performance between cost and stability makes it a reliable choice for such applications.

### 5.3. Three-Bar Truss Design Problem

**Figure 14:**
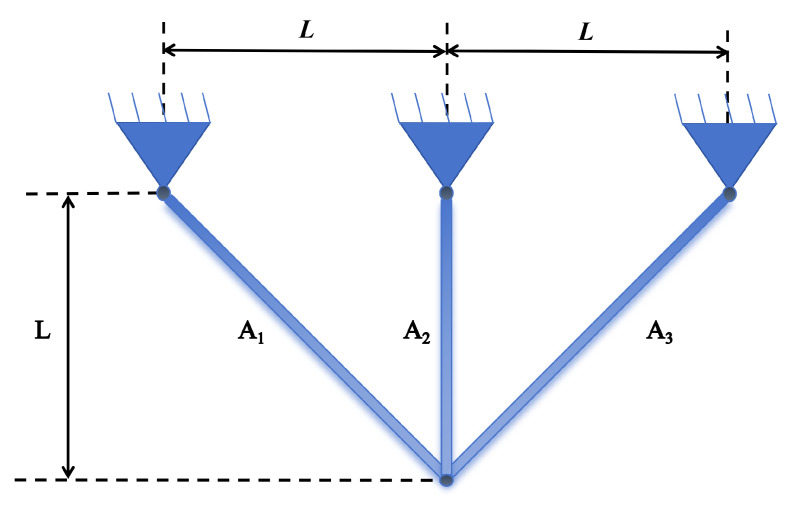
Schematic of a three-bar truss design problem.

The three-bar truss design problem serves as a widely referenced benchmark in structural optimization. The primary objective is to minimize the total volume of a truss system subject to constraints on stress, deflection, and stability under specified loading and support conditions. This problem features a simple yet representative configuration that encapsulates fundamental challenges in engineering design optimization, making it suitable for evaluating the performance of various optimization algorithms. A schematic diagram illustrating the geometric layout and loading of the three-bar truss is provided in Figure 14 [36].

The problem involves two design variables, namely, the cross-sectional areas $A_{1}$ and $A_{2}$ of the bars (denoted as $x_{1}$ and $x_{2}$). The mathematical model is given as follows:

Design variables:

(20) $$\vec{x}=\left[x_{1},x_{2}\right]=\left[A_{1},A_{2}\right],\;0\leq x_{1},x_{2}\leq 1$$

Objective function (minimize volume):

(21) $$\min f\left(\vec{x}\right)=\left(2\sqrt{2}x_{1}+x_{2}\right)\;\cdot \;l$$

Constraints (stress constraints):

(22) $$\begin{array}{cc} g_{1}\left(\vec{x}\right) & =\frac{\sqrt{2}x_{1}+x_{2}}{\sqrt{2}x_{1}^{2}+2x_{1}x_{2}}P-\sigma \leq 0,\\ g_{2}\left(\vec{x}\right) & =\frac{x_{2}}{\sqrt{2}x_{1}^{2}+2x_{1}x_{2}}P-\sigma \leq 0,\\ g_{3}\left(\vec{x}\right) & =\frac{1}{\sqrt{2}x_{2}+x_{1}}P-\sigma \leq 0, \end{array}$$

where the constants are bar length l = 100cm, external load $P\;=\;2\;{\mathrm{kN}/\mathrm{cm}}^{2}$, and allowable stress $\sigma \;=\;2\;{\mathrm{kN}/\mathrm{cm}}^{2}$.

#### 5.3.1. Structural Optimization Precision and Efficiency

**Figure 15:**
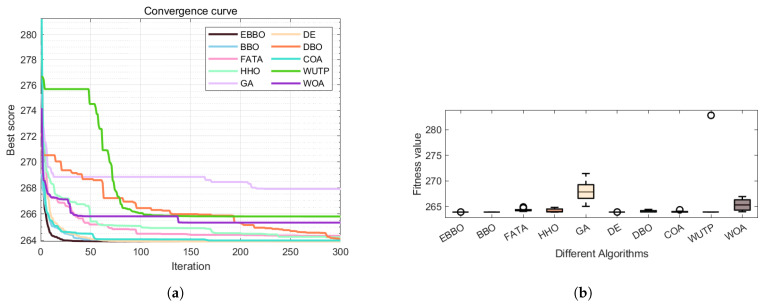
The average convergence curve and box plot of three-bar truss design problem. (**a**) The average convergence curve chart. (**b**) The box plot chart.

The three-bar truss problem tests algorithmic precision under simple constraints from Table 11 and Table 12 and Figure 15:

- EBBO, BBO, and DE all achieve costs near the theoretical optimum (∼263.896), with convergence stabilizing within 50 generations, indicating efficient and precise local search capabilities.
- GA and WOA yield inferior results (265.0052 and 263.9525, respectively) with fluctuations persisting in later iterations, reflecting insufficient exploitation efficiency or sensitivity to initial solutions.

#### 5.3.2. Solution Set Consistency and Reliability

- EBBO exhibits extremely low standard deviation (0.0003), with worst-case (263.8043) and median (263.8971) values close to optimal, indicating highly concentrated results across multiple runs.
- WUTP and GA show significantly larger standard deviations (5.9900 and 2.0025, respectively) with substantial worst-case deviations, suggesting performance variability influenced by random factors—unsuitable for engineering applications requiring stable outputs.

#### 5.3.3. Algorithm Applicability Analysis

- EBBO, BBO, and DE produce concentrated solution sets near the lower bound, meeting high-precision structural optimization requirements.
- FATA, HHO, and WUTP exhibit noticeable outliers, indicating constraint violation or search direction deviation in certain runs.

These results further validate that in structural optimization, algorithmic local exploitation capability and constraint satisfaction mechanisms directly impact final solution feasibility and economy.

### 5.4. UAV Three-Dimensional Path Planning Problem

The three-dimensional (3D) path planning problem for unmanned aerial vehicles (UAVs) serves as a critical benchmark in autonomous navigation and mission planning [37]. The primary objective is to generate a safe, flyable, and cost-effective trajectory from a start point to a destination within a complex environment characterized by terrain obstacles and threatening zones. This problem encapsulates fundamental challenges in multi-objective optimization under stringent safety constraints, making it suitable for evaluating the performance of various optimization algorithms.

The problem involves determining an ordered sequence of waypoints in 3D space. Its mathematical model is given as follows:

Path representation:

A candidate path *P* consists of *n* waypoints:

(23) $$P=p_{i}=\left(x_{i},y_{i},z_{i}\right)|i=1,2,\ldots ,n,\;\mathrm{with}\;p_{1}=S,;p_{n}=T$$

where *S* and *T* denote the start and target positions, respectively. The coordinates are bounded within the operational airspace: $0\leq x_{i},y_{i}\leq L$, and $z_{i}\geq 0$.

Objective function (minimize composite cost):

The goal is to minimize a weighted sum of five cost terms:

(24) $$\min;F\left(P\right)=\sum_{j=1}^{5}w_{j}\;\cdot \;{\tilde{J}}_{j}\left(P\right)$$

where ${\tilde{J}}_{j}$ are normalized cost terms and $w_{j}$ are corresponding weights.

1. Path length: Total Euclidean distance.
  (25) $$J_{1}\left(P\right)=\sum_{i=1}^{n-1}\left|p_{i+1}-p_{i}\right|$$
2. Threat exposure: Penalty for proximity to cylindrical threats (e.g., radars). For threat *k* with center $\left(x_{c}^{k},y_{c}^{k}\right)$, radius $r^{k}$, and height $h^{k}$:
  (26) $$J_{2}\left(P\right)=\sum_{i=1}^{n}\sum_{k=1}^{N_{t}}\Psi \left(\frac{\left(r^{k}+\Delta_{s}\right)-d_{ik}}{r^{k}+\Delta_{s}}\right)\;\cdot \;100,\;\Psi \left(a\right)=\max\left(0,a\right)$$
  where $d_{ik}=\left|\left(x_{i},y_{i}\right)-\left(x_{c}^{k},y_{c}^{k}\right)\right|$ and $\Delta_{s}=25$ m is a safety margin.
3. Path smoothness: Sum of turning angles between consecutive path segments.
  (27) $$J_{3}\left(P\right)=\sum_{i=2}^{n-1}\arccos\left(\frac{\left(p_{i}-p_{i-1}\right)\;\cdot \;\left(p_{i+1}-p_{i}\right)}{|p_{i}-p_{i-1}\left|\;\cdot \;\right|p_{i+1}-p_{i}|}\right)$$
4. Height deviation: Deviation from a desired cruising altitude $z_{\mathrm{target}}$.
  (28) $$J_{4}\left(P\right)=\left|\frac{1}{n}\sum_{i=1}^{n}z_{i}-z_{\mathrm{target}}\right|,\;z_{\mathrm{target}}=250\;m$$
5. Terrain proximity: Penalty for flying too close to the ground.
  (29) $$J_{5}\left(P\right)=\sum_{i=1}^{n}\max\left(0,;50-(z_{i}-h_{\mathrm{terrain}}\left(x_{i},y_{i}\right))\right)\;\cdot \;10$$

**Figure 16:**
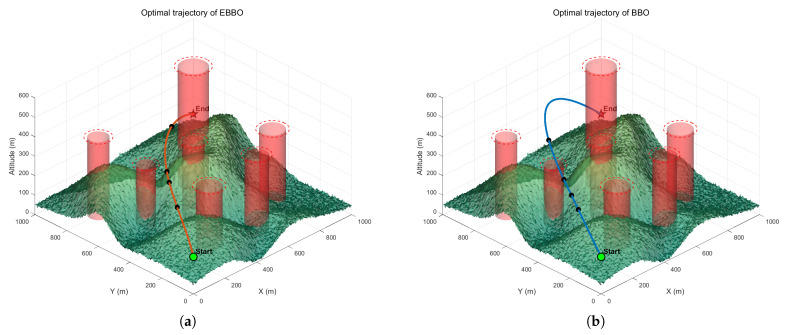
Optimal trajectory of EBBO and BBO. (**a**) Optimal trajectory of EBBO. (**b**) Optimal trajectory of BBO.

Each cost term $J_{j}$ is normalized to [0, 1] to obtain $\tilde{J}j$. The overall fitness is then adjusted by a safety bonus:

(30) $$F\left(P\right)=\frac{\sum {j=1}^{5}w_{j}\;\cdot \;\tilde{J}j\left(P\right)}{1+0.1\left(\frac{d\min^{\mathrm{threat}}}{100}+\frac{c_{\min}}{50}\right)}$$

where $d_{\min}^{\mathrm{threat}}$ is the minimum 3D distance to any threat and $c_{\min}$ is the minimum ground clearance along the path. The adopted weight vector is $w=\left[w_{1},w_{2},w_{3},w_{4},w_{5}\right]=\left[1,30,8,15,20\right]$. In the context of 3D UAV path planning within a mountainous environment, the terrain is typically represented by green elevated regions, while red zones denote obstacles or no-fly areas, as shown in Figure 16. The red columns represent obstacles or no-fly zones, and the path planning will actively avoid them and the green area represents the safe flight zone. The trajectory generated by the BBO often exhibits a longer, more tortuous path with noticeable oscillations near obstacles and irregular altitude changes, reflecting its strong exploratory behavior but lower convergence efficiency and suboptimal energy management. In contrast, the EBBO produces a significantly smoother and shorter trajectory, characterized by gradual climbs, stable avoidance of obstacles, and a more direct convergence toward the target. EBBO demonstrates superior performance in balancing exploration and exploitation, resulting in a path that is not only more energy-efficient and dynamically feasible but also maintains a safer clearance from hazardous zones, making it more suitable for practical UAV navigation in complex environments.

**Figure 17:**
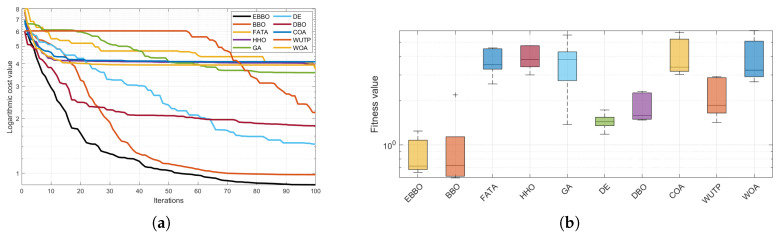
The average convergence curve and box plot of UAV 3D path planning problem. (**a**) The average convergence curve chart. (**b**) The box plot chart.

The convergence curve (Figure 17a) demonstrates that EBBO achieves the fastest convergence rate and attains the lowest logarithmic cost value in UAV 3D path planning, outperforming other metaheuristics including the original BBO. Furthermore, the box plot analysis (Figure 17b), reveals that EBBO consistently ranks first in every trial, exhibiting an exceptionally narrow interquartile range and no outliers. This indicates not only superior solution quality but also outstanding robustness and reproducibility. Collectively, these results validate EBBO as a highly efficient, reliable, and statistically dominant optimization method for autonomous UAV navigation in complex three-dimensional environments.

### 5.5. Comprehensive Performance Comparison and Mechanistic Insights

Comprehensive testing across four typical engineering optimization problems—step-cone pulley design, pressure vessel optimization, three-bar truss design, and 3D UAV path planning—demonstrates that the EBBO algorithm exhibits superior performance in solution quality, convergence speed, and stability. The solutions obtained by EBBO approach theoretical limits and demonstrate strong robustness, while BBO and DE algorithms form a well-performing second tier. In contrast, algorithms such as FATA and WOA, although capable of generating feasible solutions, suffer from issues such as significant convergence fluctuations, high dispersion in solution sets, and inadequate constraint-handling capabilities, making them unsuitable for high-reliability engineering design requirements. This study confirms that effective constraint-handling mechanisms and stable convergence characteristics are critical to the practical utility of algorithms, and that biologically consistent enhancement strategies—such as the risk-aware decision mechanism inspired by beaver behavior—provide more natural and effective ways to balance exploration and exploitation than arbitrary mathematical constructs.

## 6. Conclusions and Prospects

### 6.1. Main Conclusions

This study systematically proposes Enhanced Beaver Behavior Optimizer (EBBO) and conducts comprehensive validation across three levels: theoretical analysis, benchmark testing, and engineering applications. The following core conclusions are drawn:

1. Algorithmic enhancements are effective: Addressing deficiencies in the original BBO’s search mechanism, diversity preservation, and elite information utilization, EBBO significantly improves convergence accuracy (average objective values reduced by 15–50%), stability (standard deviations reduced by 30–70%), and statistical significance through adaptive mutation, dynamic opposition-based learning, and risk-aware decision strategy supplementation.
2. Strong engineering practicality: In three classical engineering optimization problems, EBBO consistently obtains feasible solutions approaching theoretical optima, with consistent performance across multiple independent runs, demonstrating direct applicability to real-world engineering.
3. Clear overall performance advantages.
  Compared to benchmark algorithms such as FATA, HHO, GA, DE, and WOA, EBBO demonstrates superior performance in balancing exploration and exploitation, handling constraints, and robustness in escaping local optima. This establishes a performance hierarchy with EBBO as the top tier, followed by BBO and DE as the second tier, providing an improved algorithmic choice for solving complex engineering optimization problems.
4. Biologically consistent enhancement strategy.
  Unlike generic mathematical enhancements that may disrupt algorithm coherence, EBBO’s risk-aware decision strategy maintains strong biological plausibility by directly modeling the temporal changes in beaver risk tolerance. This approach demonstrates that algorithm improvements can be both theoretically sound and biologically meaningful, providing a template for future bio-inspired algorithm development.

Furthermore, the biomimetic design of EBBO—rooted in the observable behaviors of beaver dam-building—provides not only a mechanistic analogy but also a principled framework for balancing exploration and exploitation. The role specialization (architects vs. prospectors), adaptive phase transitions, and cooperative learning mechanisms are direct translations of biological strategies that enhance algorithmic robustness and adaptability in dynamic and constrained optimization landscapes. This biologically grounded approach ensures that EBBO is not merely a heuristic hybrid but a coherent bio-inspired optimizer with explanatory power and practical efficacy.

### 6.2. Research Outlook

Although EBBO performs excellently in various tests, there remains room for further research and improvement:

1. Deepening theoretical analysis.
  The current analysis of EBBO’s convergence relies primarily on experimental validation. Future work could employ Markov chain or dynamic systems theory to provide rigorous mathematical proofs of its global convergence. Additionally, further investigation into the theoretical foundations of its parameter adaptation mechanisms could offer theoretical guidance for parameter tuning.
2. Extending the algorithmic framework.
  - Multi-objective and dynamic optimization: Currently focused on single-objective static optimization, EBBO could be extended to multi-objective optimization (MOEBBO) and dynamic environment optimization problems to broaden its applicability.
  - Hybrid parallel implementation: Integrating GPU parallel computing or distributed computing frameworks could enhance computational efficiency in high-dimensional, large-scale optimization problems.
  - Hybrid intelligent models: Exploring the integration of EBBO with machine learning methods (e.g., surrogate models, reinforcement learning) to construct hybrid optimization frameworks with learning and adaptive capabilities.
3. Expanding and deepening engineering applications.
  - More complex engineering systems: Applying EBBO to more challenging engineering domains, such as aerospace structural optimization, energy system scheduling, and intelligent manufacturing parameter optimization, to validate its effectiveness in problems with more complex constraints and multidisciplinary coupling.
  - Integration with practical systems: Investigating interfaces for integrating EBBO with CAD/CAE software to promote its seamless embedding and application in actual engineering design workflows.

In summary, as a high-performance, structurally clear metaheuristic optimization algorithm, EBBO exhibits significant potential both theoretically and practically. Through continuous theoretical refinement, framework expansion, and engineering practice, EBBO is expected to become a powerful tool for addressing complex engineering optimization problems and to provide valuable insights for the research and development of intelligent optimization algorithms.

## Figures and Tables

**Table 1:** Min, std, and avg for 20 dimensions on the CEC 2020 benchmark.

Func.	Metric	EBBO	BBO	FATA	HHO	GA	DE	DBO	COA	WUTP	WOA	LSHADE	mLSHADE- SPACMA
	min	6.14 × 10^5^	4.74 × 10^6^	1.25 × 10^11^	4.99 × 10^9^	3.93 × 10^11^	1.91 × 10^9^	2.13 × 10^11^	2.41 × 10^11^	2.06 × 10^8^	6.07 × 10^10^	5.92 × 10^5^	1.05 × 10^8^
F1	std	5.65 × 10^5^	6.98 × 10^6^	8.44 × 10^9^	1.76 × 10^9^	4.44 × 10^10^	7.76 × 10^8^	7.75 × 10^9^	1.46 × 10^10^	4.48 × 10^8^	6.98 × 10^9^	1.68 × 10^7^	1.36 × 10^8^
	avg	1.24 × 10^6^	1.12 × 10^7^	1.39 × 10^11^	7.72 × 10^9^	4.72 × 10^11^	2.86 × 10^9^	2.21 × 10^11^	2.67 × 10^11^	7.60 × 10^8^	6.85 × 10^10^	1.14 × 10^7^	2.19 × 10^8^
	min	1.14 × 10^4^	1.33 × 10^4^	3.05 × 10^4^	2.11 × 10^4^	3.08 × 10^4^	2.98 × 10^4^	2.66 × 10^4^	3.06 × 10^4^	3.14 × 10^4^	2.70 × 10^4^	3.02 × 10^4^	1.82 × 10^4^
F2	std	1.84 × 10^3^	1.09 × 10^3^	7.94 × 10^2^	1.30 × 10^3^	1.01 × 10^3^	7.64 × 10^2^	2.19 × 10^3^	9.64 × 10^2^	7.18 × 10^2^	1.21 × 10^3^	6.84 × 10^2^	1.13 × 10^3^
	avg	1.42 × 10^4^	1.52 × 10^4^	3.19 × 10^4^	2.36 × 10^4^	3.25 × 10^4^	3.15 × 10^4^	3.13 × 10^4^	3.26 × 10^4^	3.28 × 10^4^	2.81 × 10^4^	3.19 × 10^4^	2.00 × 10^4^
	min	1.33 × 10^3^	1.70 × 10^3^	3.12 × 10^3^	3.50 × 10^3^	9.99 × 10^3^	2.03 × 10^3^	3.45 × 10^3^	3.86 × 10^3^	1.81 × 10^3^	3.64 × 10^3^	1.79 × 10^3^	1.76 × 10^3^
F3	std	6.91 × 10^1^	1.41 × 10^2^	1.50 × 10^2^	1.26 × 10^2^	7.38 × 10^2^	4.49 × 10^1^	9.79 × 10^1^	8.27 × 10^1^	5.71 × 10^1^	1.31 × 10^2^	9.55 × 10^1^	1.40 × 10^2^
	avg	1.41 × 10^3^	1.90 × 10^3^	3.50 × 10^3^	3.73 × 10^3^	1.10 × 10^4^	2.12 × 10^3^	3.63 × 10^3^	4.00 × 10^3^	1.89 × 10^3^	3.77 × 10^3^	1.92 × 10^3^	1.92 × 10^3^
	min	1.96 × 10^3^	1.98 × 10^3^	1.98 × 10^6^	3.19 × 10^3^	8.31 × 10^6^	3.51 × 10^4^	1.60 × 10^7^	6.11 × 10^7^	2.02 × 10^3^	5.49 × 10^5^	2.03 × 10^3^	2.01 × 10^3^
F4	std	1.84 × 10^1^	1.26 × 10^1^	2.16 × 10^6^	7.43 × 10^3^	1.44 × 10^7^	2.39 × 10^4^	4.73 × 10^6^	4.32 × 10^7^	5.53 × 10^3^	3.43 × 10^5^	8.44 × 10^1^	1.16 × 10^2^
	avg	1.98 × 10^3^	1.99 × 10^3^	4.88 × 10^6^	1.53 × 10^4^	3.22 × 10^7^	6.90 × 10^4^	2.10 × 10^7^	1.14 × 10^8^	4.36 × 10^3^	1.06 × 10^6^	2.10 × 10^3^	2.16 × 10^3^
	min	7.84 × 10^6^	4.72 × 10^6^	1.09 × 10^8^	3.22 × 10^7^	1.52 × 10^8^	2.47 × 10^8^	1.44 × 10^8^	1.06 × 10^9^	5.46 × 10^7^	8.19 × 10^7^	2.57 × 10^6^	3.26 × 10^6^
F5	std	1.53 × 10^6^	2.37 × 10^6^	5.19 × 10^7^	1.39 × 10^7^	1.34 × 10^8^	6.38 × 10^7^	1.86 × 10^8^	4.73 × 10^8^	6.08 × 10^7^	6.60 × 10^7^	2.52 × 10^6^	3.79 × 10^6^
	avg	9.53 × 10^6^	8.82 × 10^6^	1.89 × 10^8^	5.65 × 10^7^	3.26 × 10^8^	3.67 × 10^8^	4.20 × 10^8^	1.79 × 10^9^	1.13 × 10^8^	1.56 × 10^8^	4.77 × 10^6^	7.18 × 10^6^
	min	4.36 × 10^3^	4.98 × 10^3^	1.22 × 10^4^	9.21 × 10^3^	1.36 × 10^4^	1.27 × 10^4^	1.30 × 10^4^	2.71 × 10^4^	1.02 × 10^4^	1.10 × 10^4^	7.62 × 10^3^	4.65 × 10^3^
F6	std	5.54 × 10^2^	7.12 × 10^2^	1.37 × 10^3^	9.68 × 10^2^	1.58 × 10^3^	3.45 × 10^2^	2.88 × 10^3^	3.57 × 10^3^	3.26 × 10^2^	2.94 × 10^3^	5.43 × 10^2^	7.21 × 10^2^
	avg	5.56 × 10^3^	5.76 × 10^3^	1.38 × 10^4^	1.14 × 10^4^	1.51 × 10^4^	1.31 × 10^4^	1.66 × 10^4^	3.52 × 10^4^	1.08 × 10^4^	1.68 × 10^4^	8.61 × 10^3^	5.41 × 10^3^
	min	2.94 × 10^6^	3.14 × 10^6^	3.36 × 10^7^	9.54 × 10^6^	5.37 × 10^7^	8.85 × 10^7^	1.41 × 10^8^	1.99 × 10^8^	1.80 × 10^7^	4.61 × 10^7^	2.88 × 10^6^	5.84 × 10^5^
F7	std	2.36 × 10^6^	1.91 × 10^6^	1.73 × 10^7^	8.48 × 10^6^	1.31 × 10^8^	2.50 × 10^7^	4.09 × 10^7^	7.64 × 10^7^	4.84 × 10^7^	3.17 × 10^7^	3.04 × 10^6^	1.73 × 10^6^
	avg	6.92 × 10^6^	5.48 × 10^6^	6.00 × 10^7^	2.12 × 10^7^	2.61 × 10^8^	1.26 × 10^8^	1.99 × 10^8^	3.79 × 10^8^	8.41 × 10^7^	1.11 × 10^8^	5.89 × 10^6^	3.45 × 10^6^
	min	1.33 × 10^4^	1.48 × 10^4^	3.34 × 10^4^	2.39 × 10^4^	3.44 × 10^4^	3.27 × 10^4^	2.86 × 10^4^	3.30 × 10^4^	3.39 × 10^4^	2.70 × 10^4^	3.26 × 10^4^	2.14 × 10^4^
F8	std	1.69 × 10^3^	7.82 × 10^2^	3.74 × 10^2^	1.39 × 10^3^	5.86 × 10^2^	5.30 × 10^2^	1.92 × 10^3^	9.22 × 10^2^	5.23 × 10^2^	1.49 × 10^3^	5.80 × 10^2^	2.64 × 10^3^
	avg	1.63 × 10^4^	1.65 × 10^4^	3.42 × 10^4^	2.60 × 10^4^	3.50 × 10^4^	3.36 × 10^4^	3.25 × 10^4^	3.51 × 10^4^	3.46 × 10^4^	2.96 × 10^4^	3.37 × 10^4^	2.35 × 10^4^
	min	3.57 × 10^3^	3.66 × 10^3^	6.94 × 10^3^	7.20 × 10^3^	8.77 × 10^3^	4.29 × 10^3^	7.20 × 10^3^	9.23 × 10^3^	4.24 × 10^3^	5.90 × 10^3^	3.96 × 10^3^	4.06 × 10^3^
F9	std	7.67 × 10^1^	9.90 × 10^1^	4.77 × 10^2^	3.81 × 10^2^	1.13 × 10^3^	2.10 × 10^1^	1.02 × 10^3^	8.37 × 10^2^	8.58 × 10^1^	3.92 × 10^2^	1.64 × 10^2^	1.80 × 10^2^
	avg	3.72 × 10^3^	3.85 × 10^3^	7.74 × 10^3^	7.76 × 10^3^	9.87 × 10^3^	4.33 × 10^3^	8.57 × 10^3^	1.03 × 10^4^	4.40 × 10^3^	6.47 × 10^3^	4.28 × 10^3^	4.28 × 10^3^
	min	3.36 × 10^3^	3.38 × 10^3^	6.93 × 10^3^	4.31 × 10^3^	3.80 × 10^4^	4.94 × 10^3^	1.82 × 10^4^	2.68 × 10^4^	3.71 × 10^3^	7.03 × 10^3^	3.41 × 10^3^	3.60 × 10^3^
F10	std	5.59 × 10^1^	3.89 × 10^1^	1.37 × 10^3^	1.96 × 10^2^	1.32 × 10^4^	3.86 × 10^2^	9.86 × 10^2^	1.99 × 10^3^	1.30 × 10^2^	8.14 × 10^2^	7.77 × 10^1^	1.05 × 10^2^
	avg	3.47 × 10^3^	3.43 × 10^3^	9.25 × 10^3^	4.65 × 10^3^	5.68 × 10^4^	5.55 × 10^3^	1.96 × 10^4^	2.97 × 10^4^	3.86 × 10^3^	8.20 × 10^3^	3.50 × 10^3^	3.69 × 10^3^

**Table 2:** The results of the Wilcoxon rank-sum test for 20 dimensions on the CEC 2020 benchmark.

Func.	EBBO	BBO	FATA	HHO	GA	DE	DBO	COA	WUTP	WOA	LSHADE	mLSHADE- SPACMA
F1	1	1.83 × 10^−4^	1.83 × 10^−4^	1.83 × 10^−4^	1.83 × 10^−4^	1.83 × 10^−4^	1.83 × 10^−4^	1.83 × 10^−4^	1.83 × 10^−4^	1.83 × 10^−4^	2.57 × 10^−2^	1.83 × 10^−4^
F2	1	3.07 × 10^−1^	1.83 × 10^−4^	1.83 × 10^−4^	1.83 × 10^−4^	1.83 × 10^−4^	1.83 × 10^−4^	1.83 × 10^−4^	1.83 × 10^−4^	1.83 × 10^−4^	1.83 × 10^−4^	1.83 × 10^−4^
F3	1	1.83 × 10^−4^	1.83 × 10^−4^	1.83 × 10^−4^	1.83 × 10^−4^	1.83 × 10^−4^	1.83 × 10^−4^	1.83 × 10^−4^	1.83 × 10^−4^	1.83 × 10^−4^	1.83 × 10^−4^	1.83 × 10^−4^
F4	1	2.12 × 10^−1^	1.83 × 10^−4^	1.83 × 10^−4^	1.83 × 10^−4^	1.83 × 10^−4^	1.83 × 10^−4^	1.83 × 10^−4^	1.83 × 10^−4^	1.83 × 10^−4^	1.83 × 10^−4^	2.46 × 10^−4^
F5	1	3.45 × 10^−1^	1.83 × 10^−4^	1.83 × 10^−4^	1.83 × 10^−4^	1.83 × 10^−4^	1.83 × 10^−4^	1.83 × 10^−4^	1.83 × 10^−4^	1.83 × 10^−4^	2.83 × 10^−3^	9.11 × 10^−3^
F6	1	7.91 × 10^−1^	1.83 × 10^−4^	1.83 × 10^−4^	1.83 × 10^−4^	1.83 × 10^−4^	1.83 × 10^−4^	1.83 × 10^−4^	1.83 × 10^−4^	1.83 × 10^−4^	1.83 × 10^−4^	4.27 × 10^−1^
F7	1	1.62 × 10^−1^	1.83 × 10^−4^	3.30 × 10^−4^	1.83 × 10^−4^	1.83 × 10^−4^	1.83 × 10^−4^	1.83 × 10^−4^	1.83 × 10^−4^	1.83 × 10^−4^	3.07 × 10^−1^	4.59 × 10^−3^
F8	1	8.50 × 10^−1^	1.83 × 10^−4^	1.83 × 10^−4^	1.83 × 10^−4^	1.83 × 10^−4^	1.83 × 10^−4^	1.83 × 10^−4^	1.83 × 10^−4^	1.83 × 10^−4^	1.83 × 10^−4^	1.83 × 10^−4^
F9	1	9.11 × 10^−3^	1.83 × 10^−4^	1.83 × 10^−4^	1.83 × 10^−4^	1.83 × 10^−4^	1.83 × 10^−4^	1.83 × 10^−4^	1.83 × 10^−4^	1.83 × 10^−4^	1.83 × 10^−4^	1.83 × 10^−4^
F10	1	3.12 × 10^−2^	1.83 × 10^−4^	1.83 × 10^−4^	1.83 × 10^−4^	1.83 × 10^−4^	1.83 × 10^−4^	1.83 × 10^−4^	1.83 × 10^−4^	1.83 × 10^−4^	6.23 × 10^−1^	1.83 × 10^−4^

**Table 3:** Min, std, and avg for 100 dimensions on the CEC 2017 benchmark (F1–F10).

Func.	Metric	EBBO	BBO	FATA	HHO	GA	DE	DBO	COA	WUTP	WOA	LSHADE	mLSHADE- SPACMA
	min	7.10 × 10^5^	4.73 × 10^6^	1.23 × 10^11^	5.76 × 10^9^	4.08 × 10^11^	1.93 × 10^9^	2.10 × 10^11^	2.55 × 10^11^	2.60 × 10^8^	5.46 × 10^10^	1.21 × 10^5^	8.59 × 10^7^
F1	std	1.73 × 10^5^	3.35 × 10^6^	1.07 × 10^10^	1.49 × 10^9^	6.59 × 10^10^	8.65 × 10^8^	8.44 × 10^9^	1.07 × 10^10^	4.82 × 10^8^	5.80 × 10^9^	1.82 × 10^6^	8.66 × 10^7^
	avg	8.97 × 10^5^	9.23 × 10^6^	1.37 × 10^11^	7.44 × 10^9^	4.77 × 10^11^	3.06 × 10^9^	2.20 × 10^11^	2.76 × 10^11^	8.37 × 10^8^	6.35 × 10^10^	1.73 × 10^6^	1.83 × 10^8^
	min	2.48 × 10^5^	3.03 × 10^5^	2.79 × 10^5^	2.90 × 10^5^	7.64 × 10^5^	7.13 × 10^5^	3.40 × 10^5^	3.36 × 10^5^	8.17 × 10^5^	6.26 × 10^5^	4.82 × 10^5^	1.92 × 10^5^
F3	std	3.38 × 10^4^	4.90 × 10^4^	1.72 × 10^4^	1.31 × 10^4^	9.06 × 10^4^	6.45 × 10^4^	5.52 × 10^4^	9.71 × 10^3^	1.44 × 10^5^	1.66 × 10^5^	5.67 × 10^4^	1.71 × 10^5^
	avg	3.05 × 10^5^	3.93 × 10^5^	3.08 × 10^5^	3.19 × 10^5^	9.10 × 10^5^	8.10 × 10^5^	4.01 × 10^5^	3.59 × 10^5^	9.75 × 10^5^	9.27 × 10^5^	5.55 × 10^5^	3.79 × 10^5^
	min	6.64 × 10^2^	7.10 × 10^2^	1.52 × 10^4^	1.78 × 10^3^	6.76 × 10^4^	1.46 × 10^3^	4.95 × 10^4^	9.81 × 10^4^	8.91 × 10^2^	8.62 × 10^3^	7.20 × 10^2^	9.23 × 10^2^
F4	std	5.88 × 10^1^	4.30 × 10^1^	2.82 × 10^3^	4.93 × 10^2^	3.30 × 10^4^	1.41 × 10^2^	5.24 × 10^3^	1.19 × 10^4^	2.08 × 10^2^	1.53 × 10^3^	4.61 × 10^1^	8.28 × 10^1^
	avg	7.53 × 10^2^	7.52 × 10^2^	1.85 × 10^4^	2.58 × 10^3^	1.06 × 10^5^	1.71 × 10^3^	5.84 × 10^4^	1.15 × 10^5^	1.13 × 10^3^	1.19 × 10^4^	7.96 × 10^2^	1.06 × 10^3^
	min	7.38 × 10^2^	9.56 × 10^2^	1.86 × 10^3^	1.58 × 10^3^	2.66 × 10^3^	1.59 × 10^3^	1.88 × 10^3^	2.10 × 10^3^	1.47 × 10^3^	1.67 × 10^3^	1.40 × 10^3^	8.94 × 10^2^
F5	std	5.93 × 10^1^	7.56 × 10^1^	3.48 × 10^1^	2.86 × 10^1^	1.38 × 10^2^	3.69 × 10^1^	5.89 × 10^1^	3.55 × 10^1^	3.23 × 10^1^	7.40 × 10^1^	2.94 × 10^1^	1.05 × 10^2^
	avg	8.02 × 10^2^	1.05 × 10^3^	1.90 × 10^3^	1.61 × 10^3^	2.89 × 10^3^	1.65 × 10^3^	1.94 × 10^3^	2.14 × 10^3^	1.50 × 10^3^	1.77 × 10^3^	1.44 × 10^3^	1.08 × 10^3^
	min	6.21 × 10^2^	6.36 × 10^2^	6.99 × 10^2^	6.80 × 10^2^	7.41 × 10^2^	6.16 × 10^2^	6.95 × 10^2^	7.08 × 10^2^	6.03 × 10^2^	6.92 × 10^2^	6.03 × 10^2^	6.12 × 10^2^
F6	std	5.02	4.83	2.10	3.39	1.03 × 10^1^	1.16	2.32	2.80	2.42	1.28 × 10^1^	3.50	5.02
	avg	6.28 × 10^2^	6.47 × 10^2^	7.02 × 10^2^	6.86 × 10^2^	7.57 × 10^2^	6.18 × 10^2^	6.99 × 10^2^	7.13 × 10^2^	6.07 × 10^2^	7.03 × 10^2^	6.08 × 10^2^	6.18 × 10^2^
	min	1.32 × 10^3^	1.62 × 10^3^	3.31 × 10^3^	3.56 × 10^3^	9.78 × 10^3^	2.05 × 10^3^	3.58 × 10^3^	3.80 × 10^3^	1.82 × 10^3^	3.15 × 10^3^	1.86 × 10^3^	1.59 × 10^3^
F7	std	9.46 × 10^1^	1.28 × 10^2^	1.19 × 10^2^	1.13 × 10^2^	1.03 × 10^3^	4.54 × 10^1^	8.02 × 10^1^	1.10 × 10^2^	7.30 × 10^1^	1.91 × 10^2^	6.57 × 10^1^	2.17 × 10^2^
	avg	1.46 × 10^3^	1.85 × 10^3^	3.51 × 10^3^	3.75 × 10^3^	1.12 × 10^4^	2.14 × 10^3^	3.69 × 10^3^	4.06 × 10^3^	1.91 × 10^3^	3.64 × 10^3^	1.96 × 10^3^	1.96 × 10^3^
	min	1.05 × 10^3^	1.32 × 10^3^	2.29 × 10^3^	1.89 × 10^3^	2.87 × 10^3^	1.87 × 10^3^	2.25 × 10^3^	2.58 × 10^3^	1.74 × 10^3^	2.16 × 10^3^	1.71 × 10^3^	1.18 × 10^3^
F8	std	6.50 × 10^1^	6.03 × 10^1^	7.23 × 10^1^	7.32 × 10^1^	2.82 × 10^2^	3.20 × 10^1^	5.45 × 10^1^	3.27 × 10^1^	3.96 × 10^1^	1.05 × 10^2^	4.43 × 10^1^	8.82 × 10^1^
	avg	1.16 × 10^3^	1.39 × 10^3^	2.38 × 10^3^	2.07 × 10^3^	3.18 × 10^3^	1.92 × 10^3^	2.36 × 10^3^	2.62 × 10^3^	1.80 × 10^3^	2.30 × 10^3^	1.77 × 10^3^	1.39 × 10^3^
	min	3.77 × 10^3^	1.64 × 10^4^	6.91 × 10^4^	5.82 × 10^4^	1.06 × 10^5^	4.51 × 10^4^	6.50 × 10^4^	6.50 × 10^4^	4.34 × 10^3^	5.68 × 10^4^	5.77 × 10^3^	1.37 × 10^4^
F9	std	3.34 × 10^3^	4.54 × 10^3^	3.25 × 10^3^	5.04 × 10^3^	2.20 × 10^4^	7.61 × 10^3^	6.11 × 10^3^	5.07 × 10^3^	3.91 × 10^3^	1.10 × 10^4^	4.23 × 10^3^	3.91 × 10^3^
	avg	7.87 × 10^3^	2.21 × 10^4^	7.41 × 10^4^	6.38 × 10^4^	1.38 × 10^5^	5.35 × 10^4^	7.63 × 10^4^	7.85 × 10^4^	9.52 × 10^3^	7.07 × 10^4^	1.26 × 10^4^	1.86 × 10^4^
	min	9.53 × 10^3^	1.21 × 10^4^	3.10 × 10^4^	1.93 × 10^4^	3.11 × 10^4^	3.01 × 10^4^	2.99 × 10^4^	3.19 × 10^4^	3.26 × 10^4^	2.39 × 10^4^	2.99 × 10^4^	1.79 × 10^4^
F10	std	1.39 × 10^3^	1.16 × 10^3^	6.30 × 10^2^	1.73 × 10^3^	1.00 × 10^3^	7.46 × 10^2^	1.14 × 10^3^	7.27 × 10^2^	3.97 × 10^2^	1.51 × 10^3^	6.71 × 10^2^	1.19 × 10^3^
	avg	1.28 × 10^4^	1.43 × 10^4^	3.19 × 10^4^	2.18 × 10^4^	3.25 × 10^4^	3.14 × 10^4^	3.17 × 10^4^	3.29 × 10^4^	3.33 × 10^4^	2.73 × 10^4^	3.13 × 10^4^	1.95 × 10^4^

**Table 4:** Min, std, and avg for 100 dimensions on the CEC 2017 benchmark (F11–F20).

Func.	Metric	EBBO	BBO	FATA	HHO	GA	DE	DBO	COA	WUTP	WOA	LSHADE	mLSHADE- SPACMA
	min	3.40 × 10^3^	3.51 × 10^3^	7.28 × 10^4^	4.85 × 10^4^	2.19 × 10^5^	1.22 × 10^5^	1.34 × 10^5^	1.91 × 10^5^	1.22 × 10^5^	1.03 × 10^5^	3.09 × 10^4^	5.43 × 10^3^
F11	std	9.76 × 10^2^	8.29 × 10^2^	1.22 × 10^4^	1.85 × 10^4^	7.25 × 10^4^	3.76 × 10^4^	2.02 × 10^4^	6.97 × 10^4^	6.51 × 10^4^	6.48 × 10^4^	1.13 × 10^4^	4.75 × 10^4^
	avg	5.22 × 10^3^	4.29 × 10^3^	9.13 × 10^4^	7.86 × 10^4^	3.31 × 10^5^	1.95 × 10^5^	1.57 × 10^5^	2.68 × 10^5^	2.22 × 10^5^	1.84 × 10^5^	4.85 × 10^4^	4.60 × 10^4^
	min	2.47 × 10^7^	5.26 × 10^7^	4.60 × 10^10^	7.66 × 10^8^	1.36 × 10^11^	2.51 × 10^9^	1.15 × 10^11^	1.59 × 10^11^	1.07 × 10^8^	8.88 × 10^9^	4.03 × 10^6^	7.56 × 10^7^
F12	std	1.62 × 10^7^	8.89 × 10^7^	5.46 × 10^9^	3.92 × 10^8^	5.76 × 10^10^	8.56 × 10^8^	9.52 × 10^9^	2.12 × 10^10^	5.37 × 10^7^	3.89 × 10^9^	2.36 × 10^7^	7.15 × 10^7^
	avg	5.24 × 10^7^	1.37 × 10^8^	5.45 × 10^10^	1.37 × 10^9^	2.00 × 10^11^	3.73 × 10^9^	1.31 × 10^11^	2.02 × 10^11^	1.76 × 10^8^	1.46 × 10^10^	2.80 × 10^7^	2.03 × 10^8^
	min	1.48 × 10^4^	4.33 × 10^4^	5.29 × 10^9^	1.02 × 10^7^	1.23 × 10^10^	2.67 × 10^4^	2.57 × 10^10^	4.70 × 10^10^	1.68 × 10^4^	4.04 × 10^8^	5.88 × 10^3^	3.11 × 10^4^
F13	std	6.78 × 10^3^	1.67 × 10^4^	1.41 × 10^9^	6.32 × 10^6^	1.23 × 10^10^	7.37 × 10^6^	3.25 × 10^9^	4.55 × 10^9^	2.14 × 10^5^	4.76 × 10^8^	1.43 × 10^4^	1.12 × 10^5^
	avg	2.38 × 10^4^	6.88 × 10^4^	7.81 × 10^9^	1.88 × 10^7^	2.94 × 10^10^	4.41 × 10^6^	3.04 × 10^10^	5.34 × 10^10^	1.09 × 10^5^	7.38 × 10^8^	1.52 × 10^4^	1.16 × 10^5^
	min	9.52 × 10^5^	1.38 × 10^6^	9.88 × 10^6^	3.93 × 10^6^	3.86 × 10^7^	1.99 × 10^7^	1.16 × 10^7^	3.74 × 10^7^	7.45 × 10^6^	1.09 × 10^7^	1.25 × 10^6^	1.50 × 10^5^
F14	std	9.79 × 10^5^	8.59 × 10^5^	9.31 × 10^6^	1.74 × 10^6^	5.56 × 10^7^	9.59 × 10^6^	1.01 × 10^7^	3.40 × 10^7^	1.78 × 10^7^	8.27 × 10^6^	1.51 × 10^6^	3.30 × 10^5^
	avg	2.14 × 10^6^	2.51 × 10^6^	1.98 × 10^7^	5.97 × 10^6^	8.26 × 10^7^	3.28 × 10^7^	2.22 × 10^7^	8.22 × 10^7^	2.13 × 10^7^	1.90 × 10^7^	2.60 × 10^6^	5.01 × 10^5^
	min	5.01 × 10^3^	3.06 × 10^4^	1.01 × 10^9^	3.30 × 10^6^	2.80 × 10^8^	6.26 × 10^5^	8.26 × 10^9^	1.63 × 10^10^	4.80 × 10^3^	4.71 × 10^7^	1.94 × 10^3^	4.29 × 10^3^
F15	std	1.97 × 10^3^	2.20 × 10^4^	5.58 × 10^8^	8.89 × 10^5^	2.46 × 10^9^	5.45 × 10^6^	2.03 × 10^9^	4.24 × 10^9^	1.51 × 10^5^	4.16 × 10^7^	7.92 × 10^3^	5.79 × 10^3^
	avg	8.21 × 10^3^	5.93 × 10^4^	1.87 × 10^9^	4.46 × 10^6^	3.31 × 10^9^	5.86 × 10^6^	1.04 × 10^10^	2.50 × 10^10^	7.15 × 10^4^	9.72 × 10^7^	7.03 × 10^3^	1.14 × 10^4^
	min	4.01 × 10^3^	4.57 × 10^3^	1.17 × 10^4^	7.33 × 10^3^	1.30 × 10^4^	1.06 × 10^4^	1.41 × 10^4^	2.09 × 10^4^	1.01 × 10^4^	1.30 × 10^4^	8.29 × 10^3^	4.92 × 10^3^
F16	std	7.11 × 10^2^	4.50 × 10^2^	9.88 × 10^2^	9.21 × 10^2^	4.15 × 10^3^	3.86 × 10^2^	1.11 × 10^3^	2.38 × 10^3^	3.75 × 10^2^	2.33 × 10^3^	4.90 × 10^2^	7.93 × 10^2^
	avg	5.34 × 10^3^	5.40 × 10^3^	1.31 × 10^4^	8.51 × 10^3^	1.76 × 10^4^	1.11 × 10^4^	1.53 × 10^4^	2.54 × 10^4^	1.06 × 10^4^	1.54 × 10^4^	9.05 × 10^3^	5.74 × 10^3^
	min	3.92 × 10^3^	3.79 × 10^3^	1.11 × 10^4^	5.84 × 10^3^	8.05 × 10^3^	7.40 × 10^3^	1.11 × 10^4^	1.79 × 10^6^	7.56 × 10^3^	8.54 × 10^3^	6.28 × 10^3^	4.09 × 10^3^
F17	std	6.37 × 10^2^	3.47 × 10^2^	4.28 × 10^4^	6.18 × 10^2^	2.64 × 10^4^	2.59 × 10^2^	4.94 × 10^4^	1.18 × 10^7^	1.71 × 10^2^	1.36 × 10^4^	1.97 × 10^2^	6.53 × 10^2^
	avg	4.90 × 10^3^	4.67 × 10^3^	4.38 × 10^4^	6.68 × 10^3^	2.29 × 10^4^	7.75 × 10^3^	5.66 × 10^4^	1.30 × 10^7^	7.90 × 10^3^	1.51 × 10^4^	6.64 × 10^3^	4.91 × 10^3^
	min	1.62 × 10^6^	1.19 × 10^6^	1.40 × 10^7^	1.61 × 10^6^	2.93 × 10^7^	3.54 × 10^7^	8.09 × 10^6^	1.51 × 10^8^	3.69 × 10^7^	4.85 × 10^6^	2.20 × 10^6^	2.13 × 10^5^
F18	std	1.39 × 10^6^	1.04 × 10^6^	7.51 × 10^6^	2.32 × 10^6^	6.33 × 10^7^	1.81 × 10^7^	1.33 × 10^7^	1.40 × 10^8^	1.83 × 10^7^	5.42 × 10^6^	2.22 × 10^6^	6.36 × 10^5^
	avg	3.18 × 10^6^	2.66 × 10^6^	2.74 × 10^7^	4.83 × 10^6^	9.05 × 10^7^	7.02 × 10^7^	3.12 × 10^7^	2.82 × 10^8^	6.01 × 10^7^	1.20 × 10^7^	4.60 × 10^6^	9.55 × 10^5^
	min	2.51 × 10^3^	7.42 × 10^4^	8.53 × 10^8^	1.14 × 10^7^	1.53 × 10^9^	4.56 × 10^5^	2.74 × 10^9^	1.55 × 10^10^	2.51 × 10^3^	3.28 × 10^7^	2.94 × 10^3^	3.21 × 10^3^
F19	std	2.69 × 10^3^	3.64 × 10^5^	4.44 × 10^8^	1.21 × 10^7^	1.82 × 10^9^	5.53 × 10^6^	2.86 × 10^9^	5.85 × 10^9^	5.04 × 10^3^	8.75 × 10^7^	3.45 × 10^3^	1.16 × 10^4^
	avg	5.34 × 10^3^	5.05 × 10^5^	1.54 × 10^9^	2.24 × 10^7^	3.38 × 10^9^	5.75 × 10^6^	7.42 × 10^9^	2.44 × 10^10^	8.23 × 10^3^	1.71 × 10^8^	6.47 × 10^3^	1.28 × 10^4^
	min	3.39 × 10^3^	4.30 × 10^3^	7.36 × 10^3^	5.14 × 10^3^	7.14 × 10^3^	6.95 × 10^3^	6.09 × 10^3^	7.36 × 10^3^	7.23 × 10^3^	6.14 × 10^3^	6.88 × 10^3^	5.64 × 10^3^
F20	std	7.12 × 10^2^	3.55 × 10^2^	2.06 × 10^2^	4.65 × 10^2^	6.75 × 10^2^	2.72 × 10^2^	6.05 × 10^2^	2.64 × 10^2^	2.15 × 10^2^	4.40 × 10^2^	2.05 × 10^2^	2.71 × 10^2^
	avg	4.84 × 10^3^	4.80 × 10^3^	7.67 × 10^3^	6.04 × 10^3^	8.18 × 10^3^	7.37 × 10^3^	7.33 × 10^3^	7.71 × 10^3^	7.61 × 10^3^	6.79 × 10^3^	7.16 × 10^3^	6.06 × 10^3^

**Table 5:** Min, std, and avg for 100 dimensions on the CEC 2017 benchmark (F21–F30).

Func.	Metric	EBBO	BBO	FATA	HHO	GA	DE	DBO	COA	WUTP	WOA	LSHADE	mLSHADE- SPACMA
	min	2.59 × 10^3^	2.81 × 10^3^	3.94 × 10^3^	3.82 × 10^3^	4.59 × 10^3^	3.44 × 10^3^	4.00 × 10^3^	4.71 × 10^3^	3.30 × 10^3^	3.97 × 10^3^	3.19 × 10^3^	2.71 × 10^3^
F21	std	4.74 × 10^1^	5.03 × 10^1^	9.25 × 10^1^	2.63 × 10^2^	1.64 × 10^2^	2.00 × 10^1^	1.88 × 10^2^	1.45 × 10^2^	3.90 × 10^1^	1.77 × 10^2^	5.75 × 10^1^	1.02 × 10^2^
	avg	2.68 × 10^3^	2.87 × 10^3^	4.08 × 10^3^	4.23 × 10^3^	4.94 × 10^3^	3.48 × 10^3^	4.32 × 10^3^	4.98 × 10^3^	3.38 × 10^3^	4.29 × 10^3^	3.30 × 10^3^	2.91 × 10^3^
	min	1.31 × 10^4^	1.59 × 10^4^	3.33 × 10^4^	2.34 × 10^4^	3.28 × 10^4^	3.20 × 10^4^	3.14 × 10^4^	3.30 × 10^4^	3.44 × 10^4^	2.77 × 10^4^	3.24 × 10^4^	2.07 × 10^4^
F22	std	1.71 × 10^3^	1.37 × 10^3^	5.16 × 10^2^	1.29 × 10^3^	1.19 × 10^3^	8.18 × 10^2^	1.22 × 10^3^	8.08 × 10^2^	2.38 × 10^2^	1.50 × 10^3^	6.15 × 10^2^	7.60 × 10^2^
	avg	1.58 × 10^4^	1.80 × 10^4^	3.42 × 10^4^	2.61 × 10^4^	3.46 × 10^4^	3.35 × 10^4^	3.35 × 10^4^	3.48 × 10^4^	3.48 × 10^4^	3.02 × 10^4^	3.37 × 10^4^	2.22 × 10^4^
	min	3.10 × 10^3^	3.32 × 10^3^	4.94 × 10^3^	4.76 × 10^3^	5.85 × 10^3^	3.74 × 10^3^	5.33 × 10^3^	6.19 × 10^3^	3.82 × 10^3^	4.76 × 10^3^	3.39 × 10^3^	3.27 × 10^3^
F23	std	1.09 × 10^2^	9.36 × 10^1^	2.26 × 10^2^	3.49 × 10^2^	3.91 × 10^2^	2.97 × 10^1^	2.38 × 10^2^	2.18 × 10^2^	5.96 × 10^1^	2.96 × 10^2^	1.19 × 10^2^	1.02 × 10^2^
	avg	3.23 × 10^3^	3.44 × 10^3^	5.30 × 10^3^	5.40 × 10^3^	6.34 × 10^3^	3.78 × 10^3^	5.69 × 10^3^	6.57 × 10^3^	3.91 × 10^3^	5.17 × 10^3^	3.68 × 10^3^	3.41 × 10^3^
	min	3.64 × 10^3^	3.71 × 10^3^	7.66 × 10^3^	6.73 × 10^3^	8.76 × 10^3^	4.27 × 10^3^	6.51 × 10^3^	9.56 × 10^3^	4.26 × 10^3^	5.79 × 10^3^	3.97 × 10^3^	4.01 × 10^3^
F24	std	6.08 × 10^1^	1.07 × 10^2^	1.90 × 10^2^	4.61 × 10^2^	9.76 × 10^2^	3.91 × 10^1^	1.06 × 10^3^	9.12 × 10^2^	4.94 × 10^1^	6.47 × 10^2^	1.75 × 10^2^	2.02 × 10^2^
	avg	3.71 × 10^3^	3.87 × 10^3^	7.90 × 10^3^	7.40 × 10^3^	9.92 × 10^3^	4.33 × 10^3^	8.29 × 10^3^	1.11 × 10^4^	4.34 × 10^3^	6.57 × 10^3^	4.30 × 10^3^	4.22 × 10^3^
	min	3.41 × 10^3^	3.35 × 10^3^	8.07 × 10^3^	4.22 × 10^3^	3.60 × 10^4^	5.27 × 10^3^	1.72 × 10^4^	2.86 × 10^4^	3.64 × 10^3^	7.07 × 10^3^	3.36 × 10^3^	3.49 × 10^3^
F25	std	3.50 × 10^1^	6.16 × 10^1^	1.24 × 10^3^	1.30 × 10^2^	1.70 × 10^4^	3.06 × 10^2^	1.79 × 10^3^	1.15 × 10^3^	8.69 × 10^1^	6.12 × 10^2^	8.15 × 10^1^	8.53 × 10^1^
	avg	3.45 × 10^3^	3.46 × 10^3^	9.60 × 10^3^	4.40 × 10^3^	4.93 × 10^4^	5.71 × 10^3^	1.94 × 10^4^	3.04 × 10^4^	3.74 × 10^3^	7.98 × 10^3^	3.49 × 10^3^	3.64 × 10^3^
	min	1.05 × 10^4^	3.41 × 10^3^	2.49 × 10^4^	2.80 × 10^4^	3.78 × 10^4^	1.66 × 10^4^	3.19 × 10^4^	4.78 × 10^4^	1.67 × 10^4^	3.07 × 10^4^	9.73 × 10^3^	1.37 × 10^4^
F26	std	1.85 × 10^3^	4.03 × 10^3^	4.33 × 10^3^	1.18 × 10^3^	5.14 × 10^3^	2.85 × 10^2^	4.27 × 10^3^	2.14 × 10^3^	7.47 × 10^2^	3.69 × 10^3^	2.51 × 10^3^	2.18 × 10^3^
	avg	1.28 × 10^4^	1.34 × 10^4^	3.21 × 10^4^	2.94 × 10^4^	4.47 × 10^4^	1.69 × 10^4^	3.76 × 10^4^	5.13 × 10^4^	1.80 × 10^4^	3.58 × 10^4^	1.50 × 10^4^	1.61 × 10^4^
	min	3.83 × 10^3^	3.55 × 10^3^	8.34 × 10^3^	4.56 × 10^3^	6.66 × 10^3^	4.01 × 10^3^	7.48 × 10^3^	1.36 × 10^4^	3.41 × 10^3^	4.53 × 10^3^	3.46 × 10^3^	3.59 × 10^3^
F27	std	1.10 × 10^2^	9.43 × 10^1^	6.11 × 10^2^	7.90 × 10^2^	1.34 × 10^3^	1.28 × 10^2^	9.08 × 10^2^	1.21 × 10^3^	7.64 × 10^1^	6.32 × 10^2^	9.72 × 10^1^	1.51 × 10^2^
	avg	3.95 × 10^3^	3.68 × 10^3^	9.06 × 10^3^	5.44 × 10^3^	9.77 × 10^3^	4.19 × 10^3^	8.72 × 10^3^	1.47 × 10^4^	3.54 × 10^3^	5.66 × 10^3^	3.60 × 10^3^	3.87 × 10^3^
	min	3.44 × 10^3^	3.43 × 10^3^	1.39 × 10^4^	5.00 × 10^3^	2.57 × 10^4^	7.18 × 10^3^	1.90 × 10^4^	2.80 × 10^4^	1.09 × 10^4^	9.36 × 10^3^	3.54 × 10^3^	3.72 × 10^3^
F28	std	5.31 × 10^1^	3.97 × 10^1^	1.44 × 10^3^	5.11 × 10^2^	5.30 × 10^3^	1.78 × 10^3^	2.11 × 10^3^	1.24 × 10^3^	1.84 × 10^3^	1.10 × 10^3^	6.20 × 10^1^	1.14 × 10^2^
	avg	3.55 × 10^3^	3.49 × 10^3^	1.56 × 10^4^	5.74 × 10^3^	3.64 × 10^4^	9.52 × 10^3^	2.21 × 10^4^	3.09 × 10^4^	1.51 × 10^4^	1.10 × 10^4^	3.68 × 10^3^	3.84 × 10^3^
	min	6.53 × 10^3^	6.31 × 10^3^	1.52 × 10^4^	9.00 × 10^3^	1.69 × 10^4^	9.29 × 10^3^	3.37 × 10^4^	6.93 × 10^4^	8.47 × 10^3^	1.38 × 10^4^	7.82 × 10^3^	6.44 × 10^3^
F29	std	6.39 × 10^2^	5.45 × 10^2^	2.20 × 10^3^	8.59 × 10^2^	1.57 × 10^4^	4.12 × 10^2^	2.09 × 10^4^	5.68 × 10^5^	5.37 × 10^2^	2.13 × 10^3^	6.30 × 10^2^	6.27 × 10^2^
	avg	7.52 × 10^3^	7.34 × 10^3^	1.74 × 10^4^	1.06 × 10^4^	2.57 × 10^4^	9.89 × 10^3^	5.67 × 10^4^	7.17 × 10^5^	9.51 × 10^3^	1.82 × 10^4^	9.08 × 10^3^	7.31 × 10^3^
	min	3.02 × 10^5^	3.90 × 10^6^	3.47 × 10^9^	6.73 × 10^7^	3.96 × 10^9^	2.16 × 10^6^	2.17 × 10^10^	3.79 × 10^10^	1.28 × 10^5^	1.03 × 10^9^	9.08 × 10^3^	6.73 × 10^5^
F30	std	2.95 × 10^5^	2.92 × 10^6^	1.41 × 10^9^	6.19 × 10^7^	9.37 × 10^9^	1.91 × 10^6^	2.54 × 10^9^	4.21 × 10^9^	9.59 × 10^7^	4.37 × 10^8^	3.27 × 10^4^	1.46 × 10^6^
	avg	6.20 × 10^5^	8.92 × 10^6^	5.56 × 10^9^	1.59 × 10^8^	2.00 × 10^10^	5.41 × 10^6^	2.49 × 10^10^	4.27 × 10^10^	3.08 × 10^7^	1.78 × 10^9^	3.59 × 10^4^	2.06 × 10^6^

**Table 6:** The results of the Wilcoxon rank-sum test for 100 dimensions on the CEC 2017 benchmark.

Func.	EBBO	BBO	FATA	HHO	GA	DE	DBO	COA	WUTP	WOA	LSHADE	mLSHADE- SPACMA
F1	1	1.83 × 10^−4^	1.83 × 10^−4^	1.83 × 10^−4^	1.83 × 10^−4^	1.83 × 10^−4^	1.83 × 10^−4^	1.83 × 10^−4^	1.83 × 10^−4^	1.83 × 10^−4^	9.10 × 10^−1^	1.83 × 10^−4^
F3	1	1.31 × 10^−3^	9.70 × 10^−1^	3.85 × 10^−1^	1.83 × 10^−4^	1.83 × 10^−4^	3.30 × 10^−4^	3.30 × 10^−4^	1.83 × 10^−4^	1.83 × 10^−4^	1.83 × 10^−4^	3.85 × 10^−1^
F4	1	7.91 × 10^−1^	1.83 × 10^−4^	1.83 × 10^−4^	1.83 × 10^−4^	1.83 × 10^−4^	1.83 × 10^−4^	1.83 × 10^−4^	1.83 × 10^−4^	1.83 × 10^−4^	1.04 × 10^−1^	1.83 × 10^−4^
F5	1	1.83 × 10^−4^	1.83 × 10^−4^	1.83 × 10^−4^	1.83 × 10^−4^	1.83 × 10^−4^	1.83 × 10^−4^	1.83 × 10^−4^	1.83 × 10^−4^	1.83 × 10^−4^	1.83 × 10^−4^	2.46 × 10^−4^
F6	1	2.46 × 10^−4^	1.83 × 10^−4^	1.83 × 10^−4^	1.83 × 10^−4^	1.83 × 10^−4^	1.83 × 10^−4^	1.83 × 10^−4^	1.83 × 10^−4^	1.83 × 10^−4^	1.83 × 10^−4^	1.71 × 10^−3^
F7	1	2.46 × 10^−4^	1.83 × 10^−4^	1.83 × 10^−4^	1.83 × 10^−4^	1.83 × 10^−4^	1.83 × 10^−4^	1.83 × 10^−4^	1.83 × 10^−4^	1.83 × 10^−4^	1.83 × 10^−4^	2.46 × 10^−4^
F8	1	1.83 × 10^−4^	1.83 × 10^−4^	1.83 × 10^−4^	1.83 × 10^−4^	1.83 × 10^−4^	1.83 × 10^−4^	1.83 × 10^−4^	1.83 × 10^−4^	1.83 × 10^−4^	1.83 × 10^−4^	4.40 × 10^−4^
F9	1	1.83 × 10^−4^	1.83 × 10^−4^	1.83 × 10^−4^	1.83 × 10^−4^	1.83 × 10^−4^	1.83 × 10^−4^	1.83 × 10^−4^	3.85 × 10^−1^	1.83 × 10^−4^	2.11 × 10^−2^	2.46 × 10^−4^
F10	1	1.40 × 10^−2^	1.83 × 10^−4^	1.83 × 10^−4^	1.83 × 10^−4^	1.83 × 10^−4^	1.83 × 10^−4^	1.83 × 10^−4^	1.83 × 10^−4^	1.83 × 10^−4^	1.83 × 10^−4^	1.83 × 10^−4^
F11	1	1.73 × 10^−2^	1.83 × 10^−4^	1.83 × 10^−4^	1.83 × 10^−4^	1.83 × 10^−4^	1.83 × 10^−4^	1.83 × 10^−4^	1.83 × 10^−4^	1.83 × 10^−4^	1.83 × 10^−4^	5.83 × 10^−4^
F12	1	3.61 × 10^−3^	1.83 × 10^−4^	1.83 × 10^−4^	1.83 × 10^−4^	1.83 × 10^−4^	1.83 × 10^−4^	1.83 × 10^−4^	1.83 × 10^−4^	1.83 × 10^−4^	1.40 × 10^−2^	2.46 × 10^−4^
F13	1	1.83 × 10^−4^	1.83 × 10^−4^	1.83 × 10^−4^	1.83 × 10^−4^	1.01 × 10^−3^	1.83 × 10^−4^	1.83 × 10^−4^	2.57 × 10^−2^	1.83 × 10^−4^	1.73 × 10^−2^	3.30 × 10^−4^
F14	1	4.27 × 10^−1^	1.83 × 10^−4^	1.83 × 10^−4^	1.83 × 10^−4^	1.83 × 10^−4^	1.83 × 10^−4^	1.83 × 10^−4^	1.83 × 10^−4^	1.83 × 10^−4^	3.45 × 10^−1^	3.30 × 10^−4^
F15	1	1.83 × 10^−4^	1.83 × 10^−4^	1.83 × 10^−4^	1.83 × 10^−4^	1.83 × 10^−4^	1.83 × 10^−4^	1.83 × 10^−4^	5.21 × 10^−1^	1.83 × 10^−4^	1.04 × 10^−1^	3.85 × 10^−1^
F16	1	8.50 × 10^−1^	1.83 × 10^−4^	1.83 × 10^−4^	1.83 × 10^−4^	1.83 × 10^−4^	1.83 × 10^−4^	1.83 × 10^−4^	1.83 × 10^−4^	1.83 × 10^−4^	1.83 × 10^−4^	4.27 × 10^−1^
F17	1	3.85 × 10^−1^	1.83 × 10^−4^	3.30 × 10^−4^	1.83 × 10^−4^	1.83 × 10^−4^	1.83 × 10^−4^	1.83 × 10^−4^	1.83 × 10^−4^	1.83 × 10^−4^	1.83 × 10^−4^	9.10 × 10^−1^
F18	1	5.21 × 10^−1^	1.83 × 10^−4^	1.04 × 10^−1^	1.83 × 10^−4^	1.83 × 10^−4^	1.83 × 10^−4^	1.83 × 10^−4^	1.83 × 10^−4^	3.30 × 10^−4^	1.40 × 10^−1^	4.40 × 10^−4^
F19	1	1.83 × 10^−4^	1.83 × 10^−4^	1.83 × 10^−4^	1.83 × 10^−4^	1.83 × 10^−4^	1.83 × 10^−4^	1.83 × 10^−4^	2.12 × 10^−1^	1.83 × 10^−4^	5.21 × 10^−1^	8.90 × 10^−2^
F20	1	7.91 × 10^−1^	1.83 × 10^−4^	5.83 × 10^−4^	1.83 × 10^−4^	1.83 × 10^−4^	1.83 × 10^−4^	1.83 × 10^−4^	1.83 × 10^−4^	1.83 × 10^−4^	1.83 × 10^−4^	3.30 × 10^−4^
F21	1	1.83 × 10^−4^	1.83 × 10^−4^	1.83 × 10^−4^	1.83 × 10^−4^	1.83 × 10^−4^	1.83 × 10^−4^	1.83 × 10^−4^	1.83 × 10^−4^	1.83 × 10^−4^	1.83 × 10^−4^	3.30 × 10^−4^
F22	1	1.13 × 10^−2^	1.83 × 10^−4^	1.83 × 10^−4^	1.83 × 10^−4^	1.83 × 10^−4^	1.83 × 10^−4^	1.83 × 10^−4^	1.83 × 10^−4^	1.83 × 10^−4^	1.83 × 10^−4^	1.83 × 10^−4^
F23	1	1.71 × 10^−3^	1.83 × 10^−4^	1.83 × 10^−4^	1.83 × 10^−4^	1.83 × 10^−4^	1.83 × 10^−4^	1.83 × 10^−4^	1.83 × 10^−4^	1.83 × 10^−4^	2.46 × 10^−4^	2.20 × 10^−3^
F24	1	2.20 × 10^−3^	1.83 × 10^−4^	1.83 × 10^−4^	1.83 × 10^−4^	1.83 × 10^−4^	1.83 × 10^−4^	1.83 × 10^−4^	1.83 × 10^−4^	1.83 × 10^−4^	1.83 × 10^−4^	1.83 × 10^−4^
F25	1	2.12 × 10^−1^	1.83 × 10^−4^	1.83 × 10^−4^	1.83 × 10^−4^	1.83 × 10^−4^	1.83 × 10^−4^	1.83 × 10^−4^	1.83 × 10^−4^	1.83 × 10^−4^	1.04 × 10^−1^	3.30 × 10^−4^
F26	1	1.86 × 10^−1^	1.83 × 10^−4^	1.83 × 10^−4^	1.83 × 10^−4^	1.83 × 10^−4^	1.83 × 10^−4^	1.83 × 10^−4^	1.83 × 10^−4^	1.83 × 10^−4^	4.52 × 10^−2^	5.80 × 10^−3^
F27	1	5.83 × 10^−4^	1.83 × 10^−4^	1.83 × 10^−4^	1.83 × 10^−4^	1.71 × 10^−3^	1.83 × 10^−4^	1.83 × 10^−4^	1.83 × 10^−4^	1.83 × 10^−4^	1.83 × 10^−4^	2.41 × 10^−1^
F28	1	9.11 × 10^−3^	1.83 × 10^−4^	1.83 × 10^−4^	1.83 × 10^−4^	1.83 × 10^−4^	1.83 × 10^−4^	1.83 × 10^−4^	1.83 × 10^−4^	1.83 × 10^−4^	2.83 × 10^−3^	1.83 × 10^−4^
F29	1	6.23 × 10^−1^	1.83 × 10^−4^	1.83 × 10^−4^	1.83 × 10^−4^	1.83 × 10^−4^	1.83 × 10^−4^	1.83 × 10^−4^	1.83 × 10^−4^	1.83 × 10^−4^	5.83 × 10^−4^	5.21 × 10^−1^
F30	1	1.83 × 10^−4^	1.83 × 10^−4^	1.83 × 10^−4^	1.83 × 10^−4^	1.83 × 10^−4^	1.83 × 10^−4^	1.83 × 10^−4^	7.91 × 10^−1^	1.83 × 10^−4^	1.83 × 10^−4^	1.31 × 10^−3^

**Table 7:** Step-cone pulley design best outcomes for the various algorithms.

Alg.	Optimal Values for Variables					Optimal Cost
	*l*_1_	*l*_2_	*l*_3_	*l*_4_	(*w*)	
EBBO	38.52	53.00	70.66	84.72	89.76	$1.61\;\times \;10^{1}$
BBO	38.88	53.49	71.32	85.51	90.00	$1.65\;\times \;10^{1}$
FATA	42.27	60.00	77.60	90.00	90.00	$8.80\;\times \;10^{10}$
HHO	40.34	55.52	74.01	88.74	88.74	$8.83\;\times \;10^{3}$
GA	40.06	55.12	73.49	88.11	88.07	$2.10\;\times \;10^{3}$
DE	40.83	56.18	74.90	89.79	84.86	$3.66\;\times \;10^{5}$
DBO	41.63	57.34	75.19	90.00	87.52	$2.30\;\times \;10^{10}$
COA	40.89	56.16	75.16	87.37	87.03	$3.39\;\times \;10^{10}$
WUTP	40.92	56.31	75.07	90.00	90.00	$1.83\;\times \;10^{1}$
WOA	40.54	55.79	74.38	89.18	89.11	$1.55\;\times \;10^{2}$

**Table 8:** Step-cone pulley design index statistics.

Alg.	Min	Worst	Std	Avg	Median
EBBO	1.61 × 10^1^	1.70 × 10^1^	2.94 × 10^−1^	1.66 × 10^1^	1.65 × 10^1^
BBO	1.65 × 10^1^	7.67 × 10^1^	2.27 × 10^1^	2.88 × 10^1^	1.78 × 10^1^
FATA	8.80 × 10^10^	3.13 × 10^11^	8.12 × 10^10^	1.68 × 10^11^	1.41 × 10^11^
HHO	8.83 × 10^3^	3.05 × 10^8^	1.04 × 10^8^	7.01 × 10^7^	3.29 × 10^7^
GA	2.10 × 10^3^	8.83 × 10^9^	2.77 × 10^9^	9.51 × 10^8^	9.69 × 10^6^
DE	3.66 × 10^5^	2.60 × 10^7^	9.27 × 10^6^	8.28 × 10^6^	4.42 × 10^6^
DBO	2.30 × 10^10^	5.35 × 10^11^	1.94 × 10^11^	1.56 × 10^11^	8.34 × 10^10^
COA	3.39 × 10^10^	7.91 × 10^11^	2.92 × 10^11^	3.95 × 10^11^	4.67 × 10^11^
WUTP	1.83 × 10^1^	1.57 × 10^3^	4.90 × 10^2^	1.78 × 10^2^	1.83 × 10^1^
WOA	1.55 × 10^2^	1.49 × 10^10^	5.43 × 10^9^	2.58 × 10^9^	3.10 × 10^6^

**Table 9:** Pressure vessel design best outcomes for the various algorithms.

Alg.	Optimal Values for Variables				Optimal Cost
	*x*_1_	*x*_2_	*x*_3_	*x*_4_	
EBBO	14.17	7.41	45.34	140.26	6090.60
BBO	14.99	8.03	48.58	110.07	6370.78
FATA	14.41	8.46	43.02	182.18	7008.39
HHO	16.07	7.61	51.69	86.02	6440.06
GA	19.26	9.41	58.10	45.59	7162.29
DE	14.05	7.16	44.76	146.59	6167.25
DBO	12.84	7.35	40.42	198.91	6281.37
COA	18.46	8.76	57.29	49.33	6897.19
WUTP	12.83	7.02	42.10	176.64	6059.82
WOA	14.14	7.96	43.05	165.15	6574.55

**Table 10:** Pressure vessel design index statistics.

Alg.	Min	Worst	Std	Avg	Median
EBBO	6090.60	7333.94	393.81	6712.83	6772.50
BBO	6370.78	7332.89	376.90	6709.10	6591.20
FATA	7008.39	11,114.56	1150.64	8701.61	8508.36
HHO	6440.06	8727.60	676.74	7136.17	6913.48
GA	7162.29	8522.78	467.29	7941.96	7989.01
DE	6167.25	6832.35	199.42	6417.78	6438.62
DBO	6281.37	7024.53	234.93	6510.62	6526.96
COA	6897.19	9988.41	971.11	8016.45	7740.04
WUTP	6059.82	7116.12	416.55	6363.27	6120.70
WOA	6574.55	29,292.80	6385.06	13,502.33	13,052.59

**Table 11:** Three-bar truss design problem’s best outcomes for the various algorithms.

Alg.	Optimal Values for Variables		Optimal Cost
	*A*_1_	*A*_2_	
EBBO	0.7790	0.4073	263.8958
BBO	0.7887	0.4082	263.8958
FATA	0.7980	0.3830	264.0215
HHO	0.7871	0.4128	263.8977
GA	0.8102	0.3585	265.0052
DE	0.7888	0.4079	263.8959
DBO	0.7842	0.4212	263.9178
COA	0.7885	0.4088	263.8959
WUTP	0.7891	0.4070	263.8971
WOA	0.7800	0.4333	263.9525

**Table 12:** Three-bar truss design problem index statistics.

Alg.	Min	Worst	Std	Avg	Median
EBBO	263.8958	263.8043	0.0003	263.8963	263.8971
BBO	263.8958	263.8998	0.0014	263.8969	263.8960
FATA	264.0215	264.8936	0.2691	264.3261	264.2315
HHO	263.8977	264.7968	0.3340	264.2113	264.0503
GA	265.0052	271.4398	2.0025	267.9012	267.8409
DE	263.8959	263.8991	0.0009	263.8969	263.8965
DBO	263.9178	264.4071	0.1684	264.0747	264.0271
COA	263.8959	264.3296	0.1359	263.9742	263.9147
WUTP	263.8971	282.8426	5.9900	265.7947	263.9008
WOA	263.9525	266.9285	1.1298	265.3269	265.2928

## Data Availability

The data that support the findings of this study are available from the corresponding author upon request. There are no restrictions on data availability.

## Abbreviations

The following abbreviations are used in this manuscript:

EBBO	Enhanced Beaver Behavior Optimizer
BBO	Beaver Behavior Optimizer
FATA	Fata Morgana Algorithm
HHO	Harris Hawks Optimization
GA	Genetic Algorithm
DE	Differential Evolution
DBO	Dung Beetle Optimizer
COA	Crayfish Optimization Algorithm
WUTP	Water Uptake and Transport in Plants Algorithm
WOA	Whale Optimization Algorithm
CEC	Congress on Evolutionary Computation
